# The genetically predicted causal relationship of inflammatory bowel disease with bone mineral density and osteoporosis: evidence from two-sample Mendelian randomization

**DOI:** 10.3389/fimmu.2023.1148107

**Published:** 2023-05-18

**Authors:** Dengyong Xu, Yao Chen, Xing Gao, Weidong Xie, Ya Wang, Jiaying Shen, Guang Yang, Binbin Xie

**Affiliations:** ^1^ Department of Colorectal Surgery, Sir Run Run Shaw Hospital, School of Medicine, Zhejiang University, Hangzhou, China; ^2^ Department of Medical Oncology, Sir Run Run Shaw Hospital, School of Medicine, Zhejiang University, Hangzhou, China; ^3^ Department of Oncology, The Second Affiliated Hospital of Soochow University, Suzhou, China; ^4^ Department of Gastrointestinal Surgery, The First Affiliated Hospital of Wenzhou Medical University, Wenzhou, China; ^5^ Institute of Cancer and Basic Medicine (ICBM), Chinese Academy of Sciences, Hangzhou, China; ^6^ Department of Hospital Infection-Control, Cancer Hospital of the University of Chinese Academy of Sciences, Hangzhou, China; ^7^ Department of Hospital Infection-Control, Zhejiang Cancer Hospital, Hangzhou, China; ^8^ Department of Orthopedic Surgery, The Second Affiliated Hospital of Zhejiang University, Hangzhou, China

**Keywords:** ulcerative colitis, Crohn’s disease, inflammatory bowel disease, bone mineral density, osteoporosis, Mendelian randomization

## Abstract

**Background:**

Many existing studies indicated that patients with inflammatory bowel disease (IBD), including ulcerative colitis (UC) and Crohn’s disease (CD), tend to have the risk of low total body bone mineral density (BMD), and are more likely to have osteoporosis (OS). To determine the causal relationship between IBD and bone metabolic disorders, we herein performed a two-sample Mendelian randomization analysis (TSMR) using publicly available summary statistics.

**Methods:**

Summary statistics of total body BMD, OS and IBD were downloaded from the Open Genome-Wide Association Study (GWAS), FinnGen consortium and International Inflammatory Bowel Disease Genetics Consortium (IIBDGC). The European and East Asian populations have consisted in this Mendelian Randomization (MR) work. A range of quality control procedures were taken to select eligible instrument SNPs closely associated with total body BMD, OS and IBD. To make the conclusions more reliable, we applied five robust analytical methods, among which the inverse variance weighting (IVW) method acted as the major method. Besides, heterogeneity, pleiotropy and sensitivity were evaluated.

**Results:**

In the European population, the genetic association of UC on total body BMD (OR=0.97, 95%CI=0.96,0.99, P<0.001) and overall IBD on total body BMD (OR=0.98, 95%CI=0.97,1.00, P=0.013) were significant, while the effect of CD on total body BMD was not significant enough (OR=0.99, 95%CI=0.98,1.00, P=0.085). All of UC, CD and overall IBD can be the genetic risk factor of having OS with pathological fracture (UC: OR=1.13, 95%CI=1.02,1.26, P=0.024, CD: OR=1.14, 95%CI=1.05,1.25, P=0.003, overall IBD: OR=1.13, 95%CI=1.02,1.24, P=0.015). In East Asian groups, only CD had a causal relationship with OS (OR=1.04, 95% CI=1.01,1.07, P=0.019).

**Conclusion:**

Our study revealed genetically predicted associations between IBD on total body BMD and OS in European and East Asian populations. This work supplemented the results of previous retrospective studies and demonstrated the necessity of BMD monitoring in patients with IBD.

## Introduction

Ulcerative colitis (UC) and Crohn’s disease (CD) are major forms of inflammatory bowel diseases (IBD), that mainly occurred in children and young adults (1). There is also some evidence showing that the incidence and prevalence of UC and CD in the older population are increasing in recent years (2). The characteristic of chronic and continuous inflammation of the intestines contributes to the susceptibility of colitis-associated colorectal cancer, posing a threat to the life quality and life span of IBD patients (3, 4). Besides, IBD patients are always accompanied by lower total body bone mineral density (BMD), which indicates that they are more likely to suffer from osteoporosis (OS) with or without pathological fracture or even other skeletal diseases (5–7).

The most typical features of OS are both bone quality and quantities being impaired. Meanwhile, osteoporotic fractures lead to a great disease burden in the USA with more than 25 billion dollars in predicted annual cost in 2025 (8). Fractures in some common vulnerable but important sites, represented by the hip, vertebral column and ankle, dramatically raise the risk of mortality among OS patients (9). Notably, BMD serves as a significant criterion for diagnosing and measuring the severity of OS (10). Although quantities of clues linked IBD with low total body BMD and OS, the bidirectional causal relationship between them is still ambiguous. Thus, the exploration of the genetically predicted causal relationship of IBD with BMD and OS using a novel method is urgently required.

Mendelian randomization (MR) analysis, a method of epidemiological analysis, can bolster causal inferences by employing genetic variation as an instrumental variable (IV) of the exposure factor (11). In addition to avoiding irrelevant confounders like environmental exposures, MR analysis can also lessen the impact of reverse causality, enhancing the plausibility of the causal inference (12).. In this two-sample MR work, we tried to figure out the genetically predicted causal association between IBD and skeletal diseases. Limited by the available datasets, we conducted the impact of IBD on total body BMD and OS with pathological fracture (FG) in the European population. And the impact of IBD on OS was performed in the East Asian population.

## Methods

### Study design

**Figure 1:**
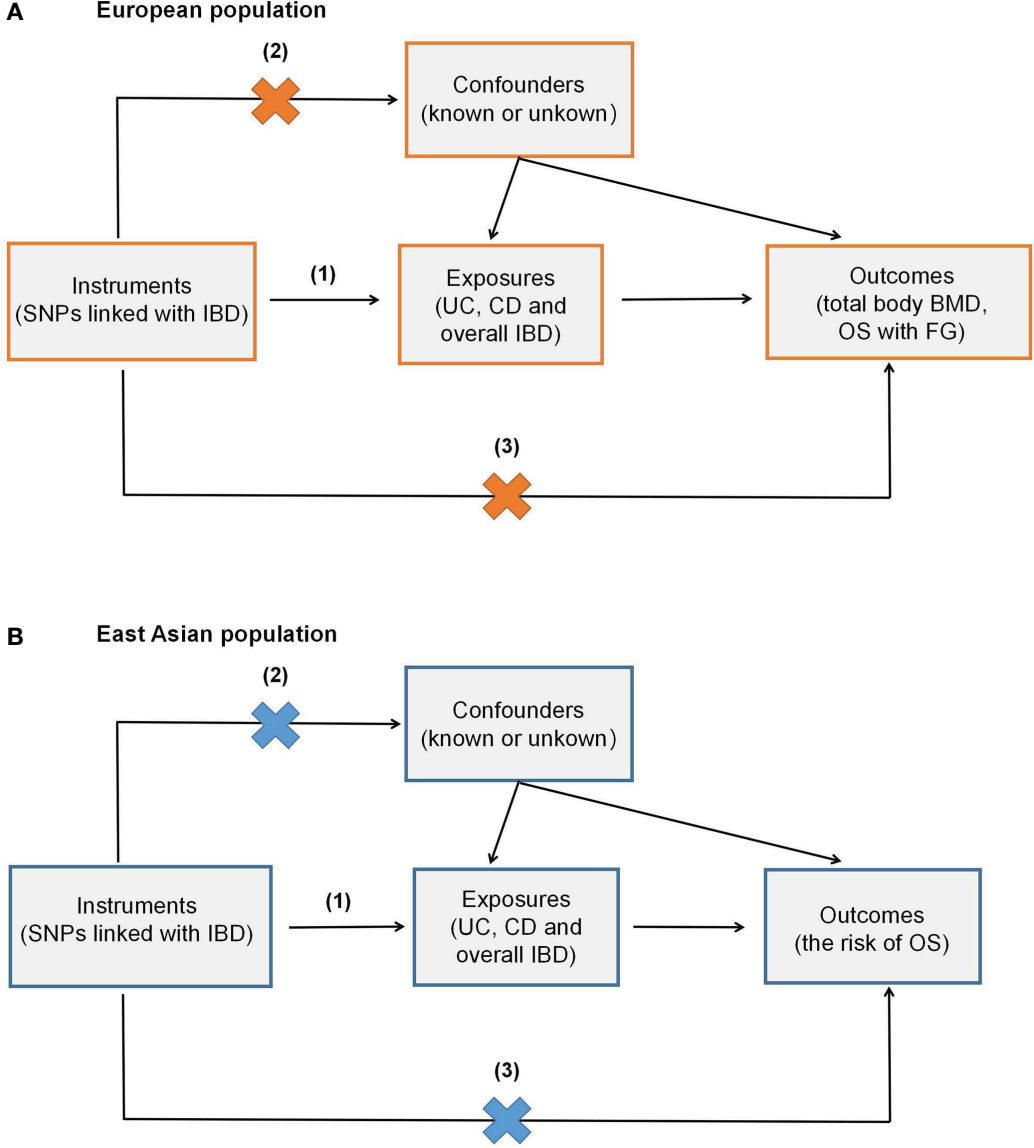
Overview of the study design. **(A)** shows the study investigating the genetic effect of IBD on total body BMD and OS with FG in the European population, while **(B)** denotes the genetic impact of IBD on OS in the East Asian population. (1) Assumption one: instrumental variables (IVs) are closely related to exposure; (2) Assumption two: IVs are independent of known or unknown confounders; (3) Assumption three: IVs only affect the outcome via exposure.

There are three main assumptions to be satisfied in the MR analysis: (1) instrumental variables (IVs) are closely related to exposure; (2) IVs are independent of any possible confounders; (3) IVs only affect the outcome through exposure (**Figure 1**). The above principles are the core of MR analysis. Two-sample MR was performed in this work, which requires two different genetic datasets to be consistent in one certain MR analysis. Of note, the data used in this work are publicly available and free to global researchers, so there was no need to provide further ethical approval and informed consent here.

### Data sources

Genetic association with the total body BMD was obtained from the meta-analysis of 30 genome-wide association studies (GWASs) of total body BMD, consisting of 56,284 European cases and 16,162,733 SNPs (13). The related summary statistics were available in the Open GWAS database (https://gwas.mrcieu.ac.uk/). Summary data of association with UC were derived from International Inflammatory Bowel Disease Genetics Consortium (IIBDGC). IIBDGC is the biggest global inflammatory bowel disease genetics database, in which European UC-associated SNPs were obtained from 13,768 cases and 33,977 controls, European CD-associated SNPs were obtained from 5,956 cases and 14,927 controls, and European overall IBD-associated SNPs were obtained from 31,665 cases and 33,977 controls (14, 15). East Asian datasets of IBD were also downloaded from IIBDGC, with 1,134 cases/3,719 controls in UC, 1,690 cases/3,719 controls in CD, and 2,824 cases/3,719 controls in overall IBD. The summary statistic of European OS with FG was gotten from the FinnGen database including 785 cases, 172,834 controls and 16,380,281 SNPs. And the data of East Asian OS were downloaded from Biobank Japan (GWAS ID: bbj-a-137; 7,788 cases/204,665 controls). To avoid genetic bias derived from ethnic differences, all the TSMR results are for the corresponding ethnicity only.

### Selection of genetic instruments

We selected the genetic instruments (IVs) according to the following inclusion criteria: (1) Single-nucleotide polymorphisms (SNPs) should be strongly associated with exposure, with the genome-wide significance level (P < 5×10^-8^); (2) Genetic variants with LD (r^2^ > 0.001) were eliminated. To clump the independence of SNPs, the linkage disequilibrium (LD) between selected SNPs was evaluated; (3) The F-statistics (beta^2^/se^2^) > 10. The F-statistics were calculated to assess the intensity of IVs. If the F-statistics of certain SNPs were less than 10 may designate less statistical power (16). All the IVs applied in this study were summarized in **Supplementary Tables 1–6**.

### Statistical analysis

In our study, two-sample Mendelian randomization (TSMR) analyses were conducted through the TwoSampleMR package (version 0.5.6) with R software (version 4.2.1) (17). After IVs being selected, the IVs’ data associated with both exposure and outcome was harmonized. The primary approach of MR analysis is the inverse-variance-weighted (IVW) method, which uses weighted regression of SNP-specific Wald ratios to evaluate the causal effects of genetically predicted exposure on outcome. Moreover, four other sensitivity assessment approaches were conducted simultaneously, namely Weighted median, MR Egger, Simple mode and Weighted mode, to test the consistency and heterogeneity of our results (18, 19). We also applied MR-PRESSO to evaluate pleiotropy and identify the outliers (20). The leave-one-out method was used to analyze the sensitivity of MR studies.

## Results

### Selected genetic instruments

We selected the IVs strictly in accordance with the criteria described above. As a result, 88, 53 and 134 independent SNPs were selected to be the IVs of European UC, CD and overall IBD respectively. And the amounts of IVs representing East Asian UC, CD and overall IBD were 10, 14 and 11 (details in **Supplementary Tables 1–6**). There was no evidence of weak instrumental bias shown by F statistics, which were also listed in the **supplementary data**.

### The causal effect of IBD on total body BMD, OS in the European population

**Figure 2:**
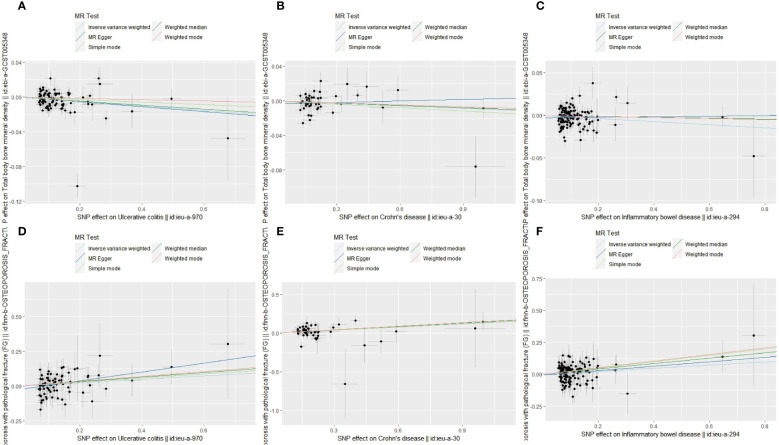
Scatter plots of MR analyses show the statistical relationship between IBD and total body BMD and the risk of OS with FG in the European population. Each dot represents an instrumental SNP. The x-axis indicates the genetic relationship with exposures (UC, CD and IBD), while the y-axis reflects the genetic association with outcomes (total body BMD and OS with FG). **(A)** UC on total body BMD: the genetically predicted UC is associated with a lower level of total body BMD; **(B)** CD on total body BMD: the genetically predicted CD is associated with a lower level of total body BMD; **(C)** overall IBD on total body BMD: the genetic predicted overall IBD is associated with a lower level of total body BMD; **(D)** UC on OS with FG: the genetic predicted UC is associated with a higher risk of OS with FG; **(E)** CD on OS with FG: the genetic predicted CD is associated with a higher risk of OS with FG; **(F)** overall IBD on OS with FG: the genetic predicted overall IBD is associated with a higher risk of OS with FG. The slope of each line shows the estimated causal effect of IBD on the bone metabolic status for each approach.

In the current MR study, outlier SNPs were excluded by the MR-PRESSO method. Therefore, the final SNPs used to conduct TSMR were less than the amounts of IVs above. The TSMR results of the IVW method and SNPs number were displayed in **Table 1**. Scatter plots were presented in **Figure 2**. All three exposures (UC, CD and IBD) demonstrated a negative genetic impact on total body BMD. However, the UC (OR=0.97, 95%CI=0.96,0.99, P<0.001) and overall IBD (OR=0.98, 95%CI=0.97,1.00, P=0.013) showed a significant association with total body BMD, while the genetic predicted effect of CD on total body BMD was not significant enough (OR=0.99, 95%CI=0.98,1.00, P=0.085). Hence, in the European population, UC and overall IBD were the causal risk factors for total body BMD.

**Table 1:** The inverse variance weighting (IVW) method results of the TSMR analyses.

Exposure	Outcome	nSNP	Beta	SE	OR	OR_lCI95	OR_uCI95	P_value
European								
UC	BMD	85	-0.026	0.008	0.974	0.959	0.990	<0.001*
CD	BMD	51	-0.010	0.006	0.990	0.979	1.001	0.085
IBD	BMD	131	-0.018	0.007	0.982	0.968	0.996	0.013*
UC	OS with FG	82	0.122	0.054	1.130	1.016	1.257	0.024*
CD	OS with FG	48	0.133	0.044	1.142	1.047	1.246	0.003*
IBD	OS with FG	129	0.120	0.049	1.128	1.024	1.242	0.015*
East Asian								
UC	OS	7	-0.008	0.032	0.992	0.932	1.056	0.801
CD	OS	14	0.038	0.016	1.038	1.006	1.072	0.019*
IBD	OS	10	0.039	0.026	1.040	0.988	1.095	0.135

An asterisk was placed if the p value of TSMR analysis was less than 0.05, which considered as significant. The BMD here refers to total body bone mineral density. SE, standard error; OR, odds ratio; CI, confidence interval; OR_lCI95, the lower 95%CI of OR; OR_uCI95, the upper 95%CI of OR; UC, ulcerative colitis; CD, Crohn’s disease; IBD, inflammatory bowel disease; OS, osteoporosis; FG, pathological fracture.

As for the genetic effect on OS with FG, 6,5 and 5 outlier SNPs in UC, CD and IBD were excluded in the following TSMR. All three exposures indicated a significant positive association with OS with FG: UC (OR=1.13, 95%CI=1.02,1.26, P=0.024), CD (OR=1.14, 95%CI=1.05,1.25, P=0.003), overall IBD (OR=1.13, 95%CI=1.02,1.24, P=0.015).

### The causal effect of IBD on OS in the East Asian population

**Figure 3:**
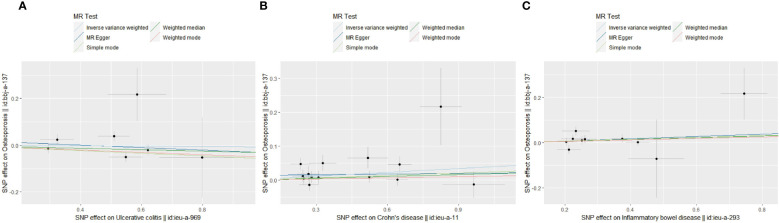
Scatter plots of MR analyses show the statistical relationship between IBD and OS in the East Asian population. Each dot represents an instrumental SNP. The x-axis indicates the genetic relationship with exposures (UC, CD and IBD), while the y-axis reflects the genetic association with outcomes (OS). **(A)** UC on OS: the genetically predicted UC is associated with a higher risk of OS; **(B)** CD on OS: the genetically predicted CD is associated with a higher risk of OS; **(C)** overall IBD on OS: the genetically predicted overall IBD is associated with a higher risk of OS. The slope of each line shows the estimated causal effect of IBD on OS for each approach.

To make clear the impact of IBD on OS in different ethnic populations, we conducted TSMR in the East Asian population. After performing the MR-PRESSO test, 7,14 and 10 SNPs were included to uncover the genetically predicted relationship of UC, CD and IBD on OS in East Asian groups. Interestingly, the results showed a significant causal effect of CD on OS (OR=1.04, 95% CI=1.01,1.07, P=0.019). While the genetic association was not significant in UC and overall IBD groups: UC on OS (OR=0.99, 95% CI=0.93,1.06, P=0.80), IBD on OS (OR=1.04, 95% CI=0.99,1.10, P=0.14). Scatter plots of IBD on OS in East Asian people were presented in **Figure 3**. Thus, we concluded that CD could serve as a genetically predicted causal risk factor of OS in the East Asian population, while UC and overall IBD could not.

### Sensitivity analysis

To approve the weak IV bias in the selected IVs of UC, CD and overall IBD, the F-statistic was calculated and ensured to be larger than 10. To validate the robustness of the above MR analyses, we performed multiplicity, heterogeneity and sensitivity analyses (details in **Supplementary Table 7**). The MR-Egger intercept and MR-PRESSO tests demonstrated no horizontal multiplicity in any of the above analyses (all P>0.05). In addition, heterogeneity was found in the heterogeneity tests of MR-Egger and IVW methods of some analyses, which are acceptable in the MR study. The results of leave-one-out sensitivity analyses indicated that the estimates of the causal effects of genetically predicted UC, CD and IBD on BMD and OS were robust (details in **Supplementary Figures 1–9**).

## Discussion

In adults with no underlying chronic disease, OS is an age-related abnormality of bone metabolism, which is most frequent in postmenopausal women (21). Nevertheless, previous retrospective studies have found that patients with IBD, consisting of children, adolescents and adults, tend to have lower BMD and are more likely to develop osteoporosis with or without pathological fractures (7). A population-based matched cohort study revealed that IBD patients have a 40% higher incidence to get fractures than people who don’t have IBD (22). Therefore, the mechanisms of how IBD affects bone metabolism is remaining a heated research topic in these decades.

In the present work, we applied TSMR analyses to find out the genetic predicted association between overall IBD (including UC and CD) and total body BMD or OS. Two different samples of each exposure and outcome which have different data structures were included to avoid possible confounding factors (23). According to our TSMR results, causal relationships differed in various ethnic groups. In the European population, UC and overall IBD acted as risk factors for total body BMD, and all three factors (UC, CD and overall IBD) had a positive causal correlation with the risk of OS accompanied by FG. As for the East Asian group, CD was the only exposure that had a positive causal relationship with OS.

There were varieties of acquired mechanisms leading to low BMD in IBD populations. Firstly, inflammations in IBD patients can be one of the most important contributors to induce bone metabolic disorders. Pro-inflammatory cytokines are engaged in the inflammation of IBD, leading to persistent gastrointestinal inflammation and tissue destruction, as well as modulating bone defects (24, 25). Secondly, nutritional deficiencies are quite common in IBD populations, which play an essential role in bone loss (26, 27). Furthermore, glucocorticoids have long been considered first-line therapeutic agents for patients with IBD (28). In addition to their conventional anti-inflammatory function, glucocorticoids have been reported to enhance intestinal epithelial barrier function (29). Hence, strategies attempting to maximize the gastrointestinal local benefits of steroids while minimizing the systemic negative effects are urgently required.

Research was increasingly focusing on the relationship between the gut microbiota and OS. It appeared that there was a bidirectional interaction between bone metabolism and gut microbiota. In rats, OS has been demonstrated to cause intestinal dysfunction. Additionally, reprogramming intestinal function through the gut-bone axis, drugs potentially modulate bone metabolism (30, 31). By producing metabolites, which enter the systemic circulation from the gastrointestinal tracts, microorganisms were known to have an impact on bone (32). Given the mystery and complexity of the gut microbiota and gut-bone axis, there are still many unknown fields to be investigated.

Previous studies reported several genetic factors which may determine bone metabolism in IBD populations. The variation of *IL-6* and *IL1-ra* genes acted as independent determinants of bone loss in patients with IBD (33). As revealed, the extent of bone loss is positively correlated with the amount of variation in specific genes mentioned above. Another gene ties to the secretion level of IL-1β, called IL1B-511*2, whose polymorphism could also be a predictor for the risk of osteopenia or osteoporosis in IBD patients (34). Low bone mass is more likely to be present in IBD among carriers of the IL1B-511*2 gene, who are also along with IL-1beta hypersecretion. Besides, a GWAS study performed in the Japanese population pointed out that the *SLC22A23* gene and the *MECOM* gene determined distinct genetic risk factors for bone loss in IBD patients from those in the general population (35). The genetic-related research above suggested that genetic variability plays an important role in bone metabolism in IBD.

According to the harmonizing results of the present MR study, we identified the SNPs with the strongest associations with total body BMD and OS, rs780094 (OR=1.03, 95%CI=1.02,1.04, P=2.54E-07) and rs34779708 (OR=8.40, 95%CI=7.56,9.34, P=1.24E-03), respectively (**Supplementary Tables 8 and 9**). Based on the search results of GWAS database, rs780094 was located on *GCKR* gene (Location: 2:27518370; Cytogenetic region:2p23.3), and rs34779708 was located on *CREM* gene (Location: 10:35177257; Cytogenetic region:10p11.21). Therefore, we targeted the known functional and disease associations of these two genes to explore whether IBD has an overlapping genetic background with BMD and OS.

cAMP responsive element modulator (*CREM*) is a gene encoding the bZIP transcription factor that binds to cAMP response elements in many viral and cellular promoters. Associated with immune system regulation, *CREM* has been implicated to play a role in various immune-mediated inflammatory processes. Aberrant expression of *CREM* was detected in patients with SLE. The expression product of *CREM* could promote the expression of inflammatory factors represented by IL-2 and IL-17 (36, 37), which in turn mediate the associated inflammatory processes. Also, *CREM* impacts T-cell activity in homeostasis and participates in the regulation of NF/κB signaling pathway (38). In addition to its role in inflammation and autoimmune diseases, Taiwanese scholars found that *CREM* expression was increased in patients with rheumatoid arthritis and its variants were involved in the progression of the disease (39). Single-cell sequencing studies showed that *CREM* regulons were most active in preosteoblast-S1 (40), implying that *CREM* variants are engaged in osteogenesis and bone homeostasis.

Glucokinase regulator (*GCKR*) gene encodes the glucokinase regulator, which is involved in metabolic modifications and is closely associated with skeletal and inflammatory phenotypes. Kasher et al. identified SNPs of the *GCKR* gene were consistently appeared in the osteoporosis phenotypes and C-reactive protein by co-localization analysis (41). The known coverage of *CREM* and *GCKR* genes reveals, to some extent, a common genetic background between IBD and bone metabolism. The effects of these loci might be diverse in UC and CD, which leads to different genetic effects observed in UC and CD subtypes.

This work is not perfect with some limitations to be mentioned here. First, based on the TSMR analysis design, this study can only provide genetic evidence for the causal relationship between IBD and OS. As a consequence, this finding can supplement earlier retrospective investigations and provide guidance for upcoming observational studies. Besides, the study was limited by the specific ethnicity. The derivation of analyses of the different ethnic populations will not be valid. It was therefore hard to generalize the result to other ethnic groups.

Some strengths also need to be highlighted. Large sample sizes in the datasets helped to reduce the bias that population stratification may cause. All of the information was gathered from reliable organizations or websites. Moreover, OS often affects older people, whereas IBD affects teenagers more frequently. Consequently, individuals with OS in IBD must be excluded from typical baseline investigations, which force applied a considerable amount of time and effort. In contrast, the current study avoided the aforementioned issues by assessing the genetic causation of the two using strongly correlated SNPs as IVs.

## Conclusion

This MR work revealed that in European population, UC and overall IBD had negative causal relationships on total body BMD, and UC, CD and overall IBD had positive causal relationships on OS with FG. While only CD showed a positive causal association with OS in East Asian population. Our results complemented former retrospective investigations and served as a reference for future animal research and clinical treatments.

## Data availability statement

The original contributions presented in the study are included in the article/**Supplementary Material**. Further inquiries can be directed to the corresponding authors.

## Author contributions

DX, XG and WX: Conceptualization and writing of the manuscript. YC and YW: Digging the data and making the tables and figures. BX, JS and GY: Review, editing and providing critical discussion. All authors contributed to the article and approved the submitted version.
