# Supplementary material for: The genetically predicted causal relationship of inflammatory bowel disease with bone mineral density and osteoporosis: evidence from two-sample Mendelian randomization
Source: Front Immunol. 2023 May 18;14:1148107. doi: 10.3389/fimmu.2023.1148107 (PMC10233018; doi:10.3389/fimmu.2023.1148107)
Supplement: Supplementary file 1 [file DataSheet_1.docx]

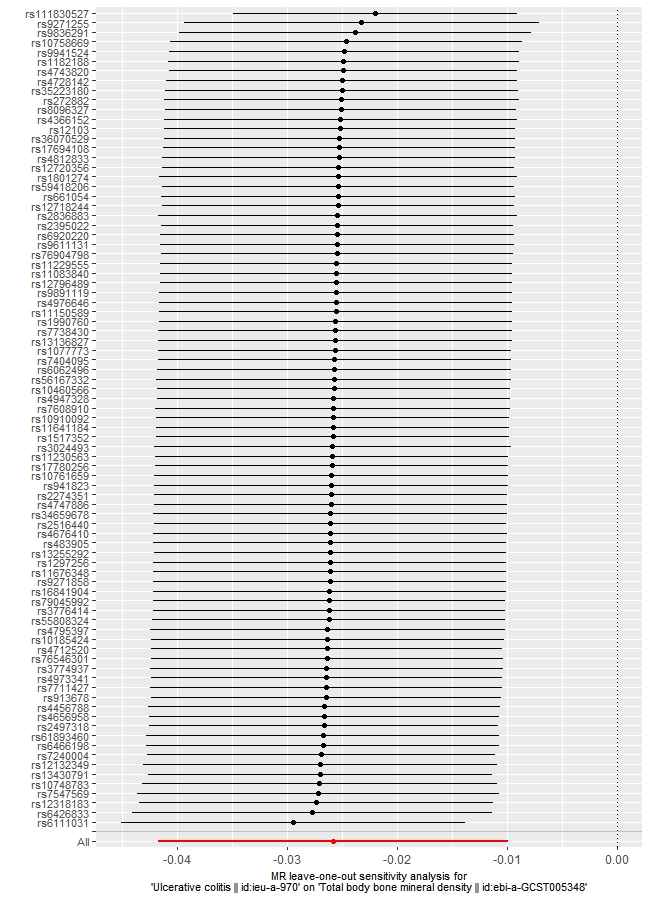


**Supplementary Figure1.** MR leave-one-out sensitivity analysis for UC on total body BMD in European population.


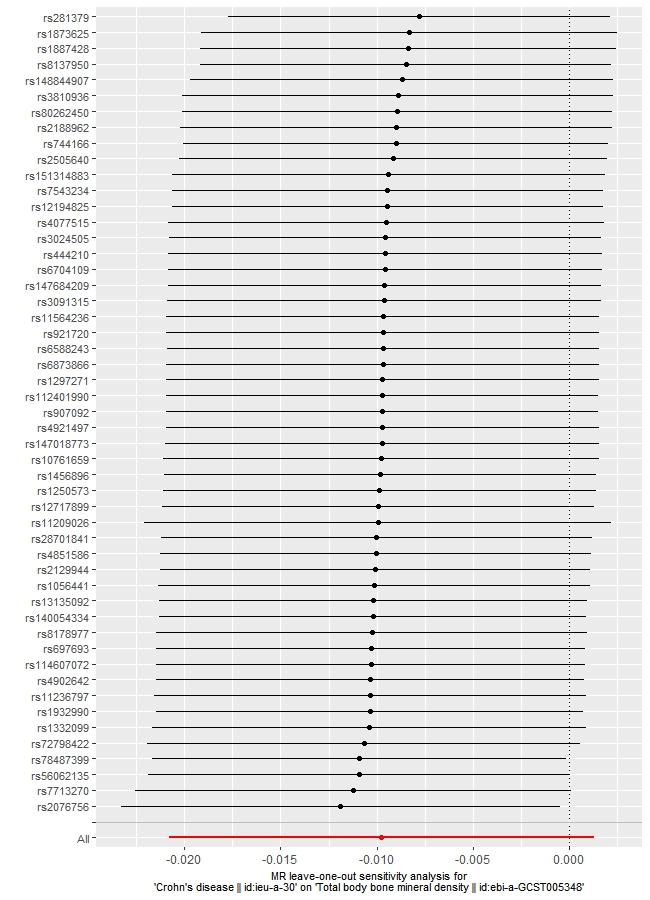


**Supplementary Figure2.** MR leave-one-out sensitivity analysis for CD on total body BMD in European population.


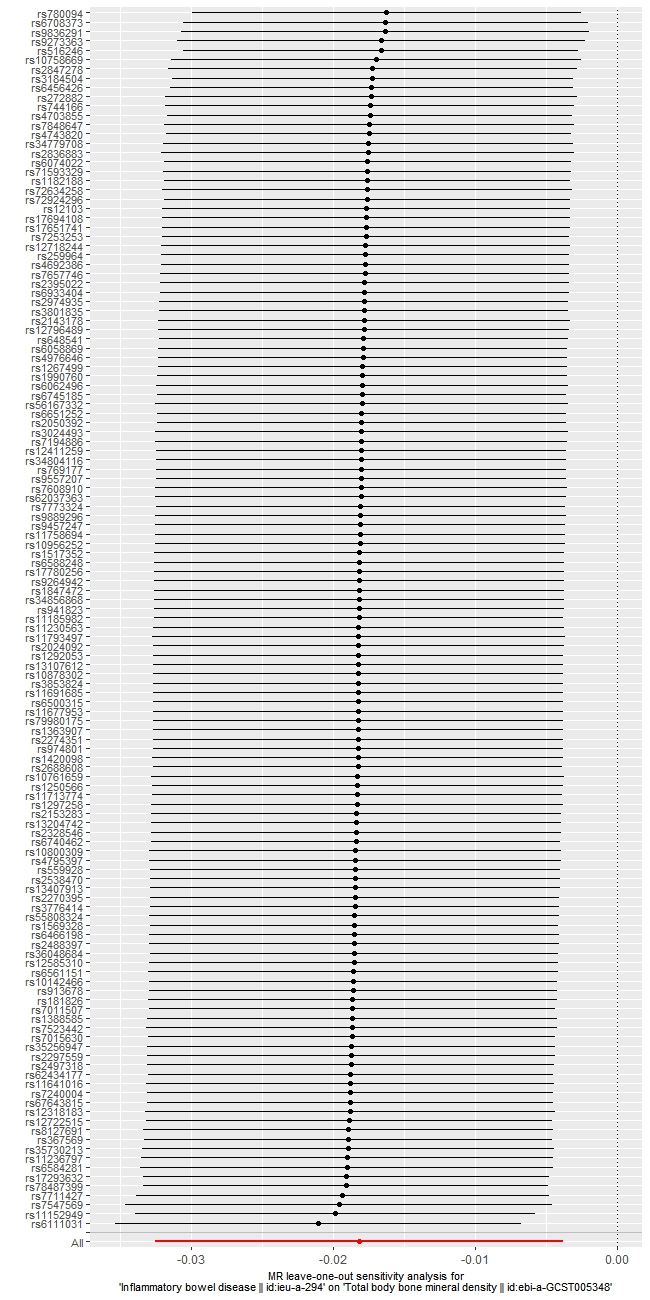


**Supplementary Figure3.** MR leave-one-out sensitivity analysis for overall IBD on total body BMD in European population.


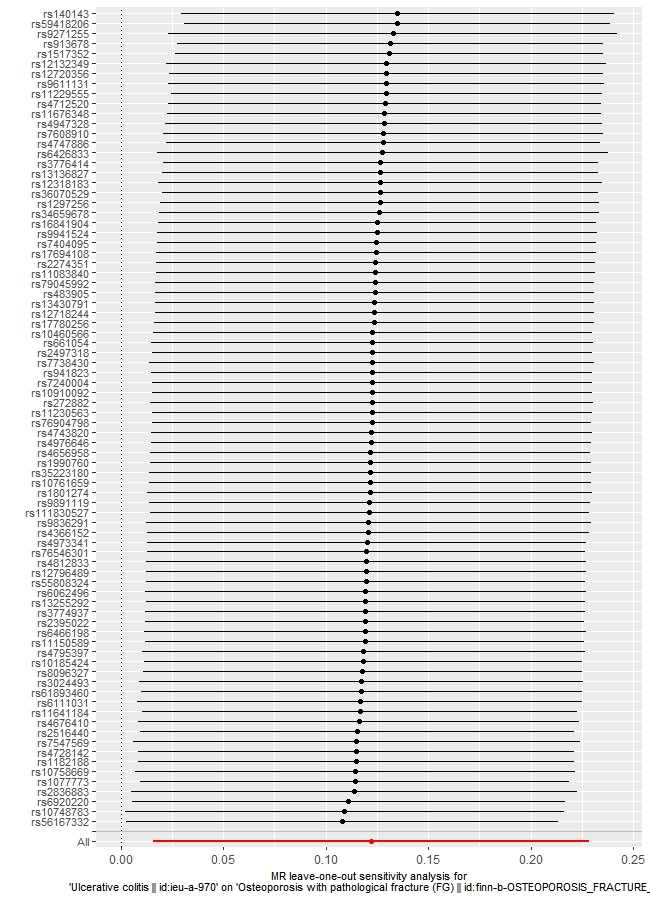


**Supplementary Figure4.** MR leave-one-out sensitivity analysis for UC on OS with FG in European population.


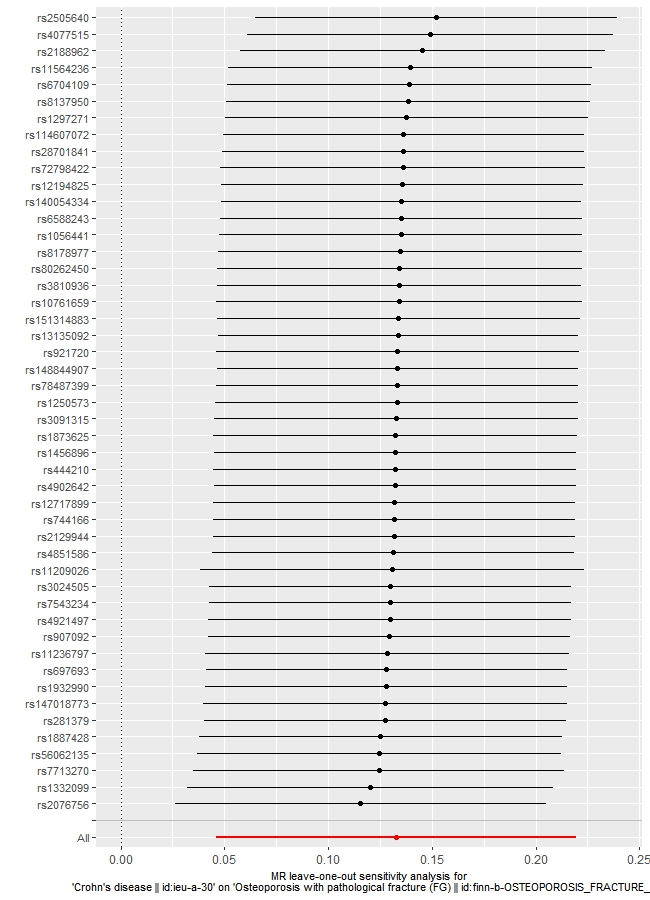


**Supplementary Figure5.** MR leave-one-out sensitivity analysis for CD on OS with FG in European population.


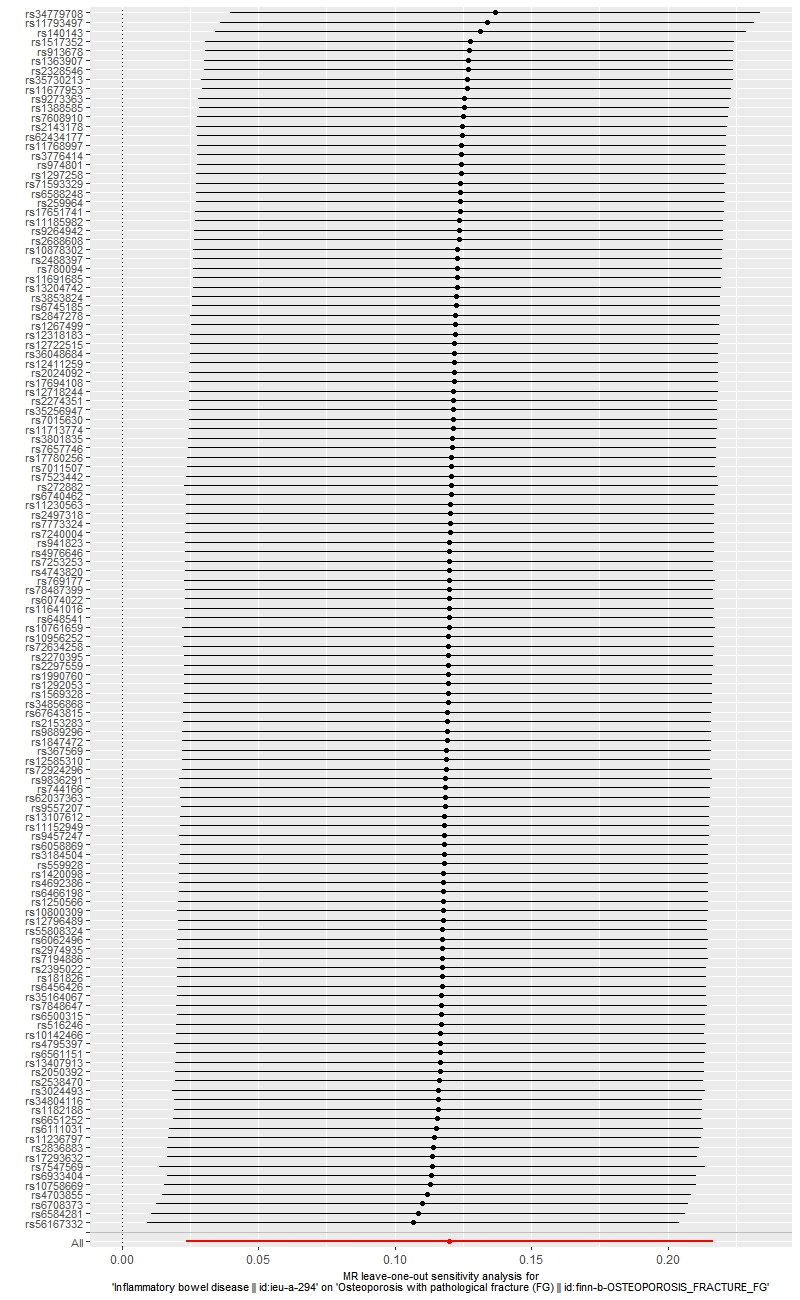


**Supplementary Figure6.** MR leave-one-out sensitivity analysis for overall IBD on OS with FG in European population.


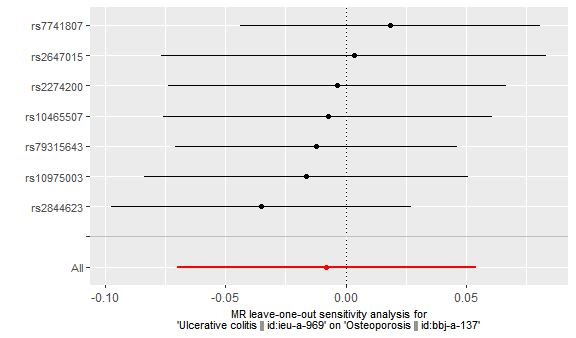


**Supplementary Figure7.** MR leave-one-out sensitivity analysis for UC on OS in East Asian population.


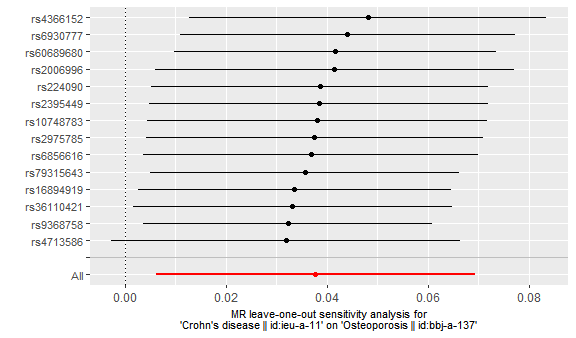


**Supplementary Figure8.** MR leave-one-out sensitivity analysis for CD on OS in East Asian population.


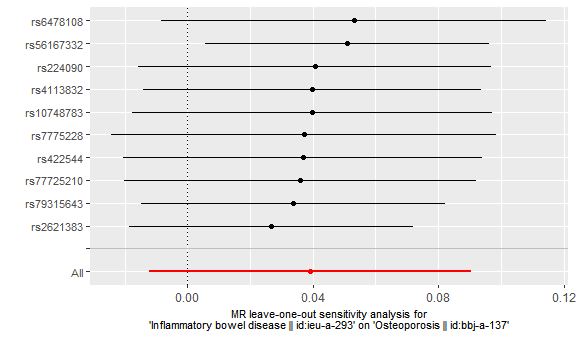


**Supplementary Figure9.** MR leave-one-out sensitivity analysis for overall IBD on OS in East Asian population.

| **exposure** | **SNP** | **effect_allele** | **other_allele** | **eaf** | **chr** | **beta** | **se** | **pval** | **F_statistic** |
| --- | --- | --- | --- | --- | --- | --- | --- | --- | --- |
| UC-European | rs12132349 | A | T | 2.81E-01 | 1 | -1.67E-01 | 1.44E-02 | 3.64E-31 | 1.35E+02 |
| UC-European | rs3024493 | A | C | 1.57E-01 | 1 | 2.26E-01 | 1.63E-02 | 1.42E-43 | 1.92E+02 |
| UC-European | rs111830527 | A | G | 5.25E-02 | 1 | -1.92E-01 | 2.93E-02 | 5.09E-11 | 4.31E+01 |
| UC-European | rs7547569 | C | T | 6.68E-02 | 1 | -4.96E-01 | 2.92E-02 | 8.71E-65 | 2.89E+02 |
| UC-European | rs12103 | C | T | 8.17E-01 | 1 | -9.96E-02 | 1.63E-02 | 9.96E-10 | 3.73E+01 |
| UC-European | rs10910092 | G | A | 4.68E-01 | 1 | -8.64E-02 | 1.28E-02 | 1.42E-11 | 4.56E+01 |
| UC-European | rs35223180 | T | G | 1.79E-01 | 1 | -1.41E-01 | 1.76E-02 | 1.04E-15 | 6.44E+01 |
| UC-European | rs6426833 | A | G | 5.36E-01 | 1 | 2.32E-01 | 1.26E-02 | 3.77E-76 | 3.41E+02 |
| UC-European | rs4656958 | G | A | 6.82E-01 | 1 | 8.24E-02 | 1.39E-02 | 2.82E-09 | 3.53E+01 |
| UC-European | rs1801274 | G | A | 4.96E-01 | 1 | -1.71E-01 | 1.27E-02 | 1.43E-41 | 1.82E+02 |
| UC-European | rs16841904 | T | C | 2.03E-01 | 1 | 8.60E-02 | 1.53E-02 | 1.90E-08 | 3.16E+01 |
| UC-European | rs7608910 | G | A | 3.91E-01 | 2 | 1.27E-01 | 1.27E-02 | 1.25E-23 | 1.00E+02 |
| UC-European | rs1990760 | T | C | 6.09E-01 | 2 | -8.56E-02 | 1.34E-02 | 1.78E-10 | 4.07E+01 |
| UC-European | rs13430791 | A | G | 1.20E-01 | 2 | 1.06E-01 | 1.87E-02 | 1.39E-08 | 3.22E+01 |
| UC-European | rs9941524 | G | A | 4.56E-01 | 2 | 9.77E-02 | 1.28E-02 | 2.15E-14 | 5.84E+01 |
| UC-European | rs10460566 | A | G | 7.61E-01 | 2 | -8.18E-02 | 1.45E-02 | 1.60E-08 | 3.19E+01 |
| UC-European | rs4973341 | T | C | 6.63E-01 | 2 | 7.35E-02 | 1.31E-02 | 2.25E-08 | 3.13E+01 |
| UC-European | rs10185424 | G | T | 5.40E-01 | 2 | -9.66E-02 | 1.26E-02 | 1.47E-14 | 5.91E+01 |
| UC-European | rs1517352 | C | A | 6.05E-01 | 2 | 7.78E-02 | 1.30E-02 | 2.10E-09 | 3.59E+01 |
| UC-European | rs4676410 | A | G | 2.04E-01 | 2 | 1.42E-01 | 1.57E-02 | 1.85E-19 | 8.14E+01 |
| UC-European | rs11676348 | T | C | 4.76E-01 | 2 | 7.44E-02 | 1.24E-02 | 2.08E-09 | 3.59E+01 |
| UC-European | rs9836291 | A | G | 2.88E-01 | 3 | 1.70E-01 | 1.32E-02 | 8.20E-38 | 1.65E+02 |
| UC-European | rs13136827 | C | T | 1.62E-01 | 4 | -1.12E-01 | 1.76E-02 | 2.35E-10 | 4.02E+01 |
| UC-European | rs3774937 | C | T | 3.26E-01 | 4 | 9.93E-02 | 1.32E-02 | 4.61E-14 | 5.69E+01 |
| UC-European | rs56167332 | A | C | 3.38E-01 | 5 | 1.41E-01 | 1.32E-02 | 7.27E-27 | 1.15E+02 |
| UC-European | rs272882 | T | G | 6.73E-01 | 5 | 1.46E-01 | 1.39E-02 | 6.67E-26 | 1.11E+02 |
| UC-European | rs36070529 | A | G | 2.00E-01 | 5 | -9.17E-02 | 1.60E-02 | 1.04E-08 | 3.28E+01 |
| UC-European | rs4976646 | C | T | 3.42E-01 | 5 | 7.88E-02 | 1.32E-02 | 2.52E-09 | 3.55E+01 |
| UC-European | rs3776414 | G | T | 3.76E-01 | 5 | 7.05E-02 | 1.28E-02 | 4.10E-08 | 3.01E+01 |
| UC-European | rs7711427 | C | A | 6.13E-01 | 5 | 8.89E-02 | 1.28E-02 | 3.67E-12 | 4.83E+01 |
| UC-European | rs7738430 | C | T | 2.62E-02 | 6 | 3.68E-01 | 3.41E-02 | 3.51E-27 | 1.17E+02 |
| UC-European | rs4947328 | G | A | 2.37E-02 | 6 | 2.39E-01 | 3.81E-02 | 3.38E-10 | 3.94E+01 |
| UC-European | rs9271858 | G | A | 5.11E-01 | 6 | 1.23E-01 | 1.29E-02 | 2.42E-21 | 9.00E+01 |
| UC-European | rs4712520 | C | T | 8.18E-01 | 6 | 9.30E-02 | 1.66E-02 | 2.21E-08 | 3.13E+01 |
| UC-European | rs2516440 | A | G | 3.22E-01 | 6 | -9.98E-02 | 1.38E-02 | 4.40E-13 | 5.25E+01 |
| UC-European | rs9271255 | T | C | 7.32E-01 | 6 | -2.85E-01 | 1.38E-02 | 1.31E-94 | 4.26E+02 |
| UC-European | rs34659678 | T | C | 5.73E-02 | 6 | 2.10E-01 | 2.51E-02 | 5.95E-17 | 7.00E+01 |
| UC-European | rs6920220 | A | G | 2.09E-01 | 6 | 1.47E-01 | 1.52E-02 | 4.78E-22 | 9.32E+01 |
| UC-European | rs1077773 | A | G | 5.24E-01 | 7 | 7.21E-02 | 1.24E-02 | 5.96E-09 | 3.38E+01 |
| UC-European | rs12718244 | A | G | 4.08E-01 | 7 | 7.18E-02 | 1.27E-02 | 1.41E-08 | 3.22E+01 |
| UC-European | rs1182188 | C | T | 2.99E-01 | 7 | -1.08E-01 | 1.38E-02 | 5.03E-15 | 6.12E+01 |
| UC-European | rs2395022 | C | A | 9.59E-01 | 7 | -1.84E-01 | 2.92E-02 | 2.88E-10 | 3.98E+01 |
| UC-European | rs4728142 | A | G | 4.39E-01 | 7 | 9.70E-02 | 1.27E-02 | 1.92E-14 | 5.86E+01 |
| UC-European | rs76546301 | A | G | 1.82E-02 | 7 | 2.65E-01 | 4.10E-02 | 1.05E-10 | 4.17E+01 |
| UC-European | rs6466198 | T | A | 3.86E-01 | 7 | 1.34E-01 | 1.28E-02 | 1.90E-25 | 1.09E+02 |
| UC-European | rs13255292 | T | C | 3.28E-01 | 8 | -7.55E-02 | 1.37E-02 | 3.82E-08 | 3.02E+01 |
| UC-European | rs4366152 | C | T | 6.80E-01 | 9 | 1.20E-01 | 1.36E-02 | 7.79E-19 | 7.86E+01 |
| UC-European | rs10870077 | G | C | 5.72E-01 | 9 | -1.36E-01 | 1.26E-02 | 5.77E-27 | 1.16E+02 |
| UC-European | rs10758669 | A | C | 6.50E-01 | 9 | -1.43E-01 | 1.29E-02 | 1.04E-28 | 1.24E+02 |
| UC-European | rs4743820 | T | C | 7.02E-01 | 9 | 8.09E-02 | 1.38E-02 | 4.05E-09 | 3.46E+01 |
| UC-European | rs10748783 | A | C | 5.24E-01 | 10 | -1.65E-01 | 1.26E-02 | 7.73E-39 | 1.70E+02 |
| UC-European | rs2274351 | T | C | 5.37E-01 | 10 | 7.11E-02 | 1.30E-02 | 4.90E-08 | 2.98E+01 |
| UC-European | rs2497318 | T | C | 4.50E-01 | 10 | -7.14E-02 | 1.25E-02 | 1.15E-08 | 3.26E+01 |
| UC-European | rs4747886 | T | C | 4.08E-01 | 10 | 7.38E-02 | 1.29E-02 | 9.58E-09 | 3.29E+01 |
| UC-European | rs59418206 | A | G | 3.51E-01 | 10 | 7.36E-02 | 1.30E-02 | 1.45E-08 | 3.21E+01 |
| UC-European | rs10761659 | G | A | 5.40E-01 | 10 | 1.17E-01 | 1.26E-02 | 1.50E-20 | 8.64E+01 |
| UC-European | rs12796489 | A | C | 2.29E-02 | 11 | -6.76E-01 | 5.60E-02 | 1.22E-33 | 1.46E+02 |
| UC-European | rs61893460 | A | G | 4.45E-01 | 11 | 1.21E-01 | 1.25E-02 | 4.60E-22 | 9.33E+01 |
| UC-European | rs11229555 | T | G | 2.52E-01 | 11 | -8.23E-02 | 1.45E-02 | 1.21E-08 | 3.25E+01 |
| UC-European | rs11230563 | T | C | 3.48E-01 | 11 | -7.51E-02 | 1.34E-02 | 1.90E-08 | 3.16E+01 |
| UC-European | rs661054 | G | A | 3.41E-01 | 11 | -1.25E-01 | 1.36E-02 | 3.18E-20 | 8.49E+01 |
| UC-European | rs483905 | A | G | 2.89E-01 | 11 | 8.50E-02 | 1.35E-02 | 3.16E-10 | 3.96E+01 |
| UC-European | rs12318183 | A | C | 3.85E-01 | 12 | 1.62E-01 | 1.27E-02 | 1.44E-37 | 1.64E+02 |
| UC-European | rs76904798 | T | C | 1.37E-01 | 12 | 1.05E-01 | 1.76E-02 | 2.78E-09 | 3.53E+01 |
| UC-European | rs941823 | C | T | 7.51E-01 | 13 | 1.09E-01 | 1.47E-02 | 1.39E-13 | 5.47E+01 |
| UC-European | rs1927681 | A | T | 4.43E-01 | 13 | -4.79E-01 | 1.27E-02 | 1.00E-200 | 1.43E+03 |
| UC-European | rs55808324 | A | G | 9.32E-02 | 14 | 1.27E-01 | 2.10E-02 | 1.47E-09 | 3.66E+01 |
| UC-European | rs7404095 | C | T | 5.80E-01 | 16 | 7.18E-02 | 1.27E-02 | 1.52E-08 | 3.20E+01 |
| UC-European | rs79045992 | A | G | 1.03E-01 | 16 | 1.18E-01 | 2.08E-02 | 1.43E-08 | 3.21E+01 |
| UC-European | rs11150589 | C | T | 5.27E-01 | 16 | -7.99E-02 | 1.27E-02 | 3.28E-10 | 3.95E+01 |
| UC-European | rs11641184 | A | C | 4.76E-01 | 16 | 7.80E-02 | 1.25E-02 | 4.24E-10 | 3.90E+01 |
| UC-European | rs4795397 | G | A | 4.71E-01 | 17 | 1.40E-01 | 1.26E-02 | 1.01E-28 | 1.24E+02 |
| UC-European | rs9891119 | C | A | 3.54E-01 | 17 | -8.95E-02 | 1.33E-02 | 1.72E-11 | 4.53E+01 |
| UC-European | rs17780256 | C | A | 1.93E-01 | 17 | -1.15E-01 | 1.60E-02 | 6.13E-13 | 5.18E+01 |
| UC-European | rs7240004 | G | A | 3.80E-01 | 18 | -8.24E-02 | 1.30E-02 | 2.50E-10 | 4.00E+01 |
| UC-European | rs8096327 | G | A | 3.84E-01 | 18 | 9.38E-02 | 1.28E-02 | 2.24E-13 | 5.38E+01 |
| UC-European | rs17694108 | A | G | 2.80E-01 | 19 | 9.58E-02 | 1.39E-02 | 6.17E-12 | 4.73E+01 |
| UC-European | rs12720356 | C | A | 8.57E-02 | 19 | 1.53E-01 | 2.28E-02 | 1.67E-11 | 4.53E+01 |
| UC-European | rs11083840 | G | T | 4.03E-01 | 19 | 6.91E-02 | 1.25E-02 | 3.41E-08 | 3.05E+01 |
| UC-European | rs6111031 | T | C | 1.59E-01 | 20 | -2.61E-01 | 1.91E-02 | 1.33E-42 | 1.87E+02 |
| UC-European | rs6062496 | A | G | 5.69E-01 | 20 | 1.14E-01 | 1.29E-02 | 9.14E-19 | 7.82E+01 |
| UC-European | rs913678 | C | T | 3.29E-01 | 20 | -7.58E-02 | 1.33E-02 | 1.23E-08 | 3.24E+01 |
| UC-European | rs4812833 | A | G | 5.19E-01 | 20 | 1.03E-01 | 1.26E-02 | 1.87E-16 | 6.77E+01 |
| UC-European | rs1297256 | T | C | 4.25E-01 | 21 | -1.01E-01 | 1.27E-02 | 2.10E-15 | 6.30E+01 |
| UC-European | rs4456788 | A | G | 6.11E-01 | 21 | -1.03E-01 | 1.27E-02 | 7.07E-16 | 6.51E+01 |
| UC-European | rs2836883 | A | G | 2.73E-01 | 21 | -2.27E-01 | 1.47E-02 | 1.47E-53 | 2.37E+02 |
| UC-European | rs9611131 | C | T | 1.48E-01 | 22 | -1.43E-01 | 1.82E-02 | 3.84E-15 | 6.18E+01 |
| UC-European | rs140143 | T | G | 3.90E-01 | 22 | -1.30E-01 | 1.43E-02 | 1.19E-19 | 8.23E+01 |

**Supplementary Table1.** SNPs selected as IVs for UC in European population.

| **exposure** | **SNP** | **effect_allele** | **other_allele** | **eaf** | **chr** | **beta** | **se** | **pval** | **F_statistic** |
| --- | --- | --- | --- | --- | --- | --- | --- | --- | --- |
| CD-European | rs6588243 | C | A | 5.90E-01 | 1 | 1.32E-01 | 2.34E-02 | 1.78E-08 | 3.17E+01 |
| CD-European | rs11209026 | A | G | 5.63E-02 | 1 | -9.95E-01 | 6.39E-02 | 1.05E-54 | 2.43E+02 |
| CD-European | rs6704109 | T | C | 2.56E-01 | 1 | 2.02E-01 | 2.56E-02 | 2.77E-15 | 6.23E+01 |
| CD-European | rs697693 | A | G | 2.01E-01 | 1 | 1.72E-01 | 2.81E-02 | 8.36E-10 | 3.76E+01 |
| CD-European | rs7543234 | T | C | 2.39E-01 | 1 | 1.55E-01 | 2.67E-02 | 6.10E-09 | 3.39E+01 |
| CD-European | rs3024505 | A | G | 1.62E-01 | 1 | 1.78E-01 | 3.02E-02 | 3.90E-09 | 3.47E+01 |
| CD-European | rs12692254 | T | A | 5.43E-01 | 2 | 3.01E-01 | 2.32E-02 | 1.86E-38 | 1.69E+02 |
| CD-European | rs78487399 | C | G | 1.01E-01 | 2 | 2.26E-01 | 3.70E-02 | 1.03E-09 | 3.73E+01 |
| CD-European | rs112401990 | A | G | 3.73E-01 | 2 | 1.32E-01 | 2.37E-02 | 2.35E-08 | 3.11E+01 |
| CD-European | rs4851586 | C | T | 7.60E-01 | 2 | -1.69E-01 | 2.61E-02 | 9.94E-11 | 4.19E+01 |
| CD-European | rs1873625 | A | C | 3.20E-01 | 3 | 1.81E-01 | 2.43E-02 | 1.09E-13 | 5.53E+01 |
| CD-European | rs13135092 | G | A | 9.54E-02 | 4 | 2.21E-01 | 3.89E-02 | 1.21E-08 | 3.24E+01 |
| CD-European | rs7713270 | T | C | 6.24E-01 | 5 | 2.97E-01 | 2.41E-02 | 6.97E-35 | 1.51E+02 |
| CD-European | rs2188962 | T | C | 4.40E-01 | 5 | 2.12E-01 | 2.28E-02 | 1.36E-20 | 8.68E+01 |
| CD-European | rs147018773 | T | C | 9.67E-02 | 5 | 3.22E-01 | 3.75E-02 | 8.89E-18 | 7.36E+01 |
| CD-European | rs4921497 | G | C | 3.29E-01 | 5 | 1.60E-01 | 2.44E-02 | 5.49E-11 | 4.32E+01 |
| CD-European | rs6873866 | C | T | 5.35E-01 | 5 | -1.68E-01 | 2.39E-02 | 2.06E-12 | 4.95E+01 |
| CD-European | rs12717899 | T | G | 7.94E-01 | 5 | 1.59E-01 | 2.89E-02 | 3.59E-08 | 3.03E+01 |
| CD-European | rs12194825 | A | T | 1.86E-01 | 6 | -1.72E-01 | 2.98E-02 | 8.00E-09 | 3.33E+01 |
| CD-European | rs148844907 | A | T | 8.28E-03 | 6 | 9.58E-01 | 1.42E-01 | 1.47E-11 | 4.56E+01 |
| CD-European | rs114607072 | T | G | 4.03E-02 | 6 | 4.42E-01 | 6.29E-02 | 2.20E-12 | 4.93E+01 |
| CD-European | rs28701841 | A | G | 1.17E-01 | 6 | 2.24E-01 | 3.73E-02 | 1.85E-09 | 3.62E+01 |
| CD-European | rs140054334 | T | C | 3.83E-02 | 6 | 3.50E-01 | 6.28E-02 | 2.57E-08 | 3.10E+01 |
| CD-European | rs444210 | G | A | 5.47E-01 | 6 | 1.63E-01 | 2.29E-02 | 1.02E-12 | 5.09E+01 |
| CD-European | rs1456896 | T | C | 6.98E-01 | 7 | 1.39E-01 | 2.51E-02 | 2.90E-08 | 3.08E+01 |
| CD-European | rs921720 | G | A | 6.19E-01 | 8 | 1.63E-01 | 2.37E-02 | 6.40E-12 | 4.72E+01 |
| CD-European | rs1887428 | C | G | 6.23E-01 | 9 | -1.68E-01 | 2.43E-02 | 4.22E-12 | 4.79E+01 |
| CD-European | rs3810936 | C | T | 6.98E-01 | 9 | 2.08E-01 | 2.63E-02 | 2.46E-15 | 6.24E+01 |
| CD-European | rs4077515 | T | C | 4.20E-01 | 9 | 2.16E-01 | 2.35E-02 | 4.37E-20 | 8.44E+01 |
| CD-European | rs1250573 | A | G | 2.87E-01 | 10 | -1.71E-01 | 2.64E-02 | 9.01E-11 | 4.19E+01 |
| CD-European | rs10761659 | G | A | 5.53E-01 | 10 | 2.12E-01 | 2.37E-02 | 3.42E-19 | 8.00E+01 |
| CD-European | rs2505640 | G | A | 6.44E-01 | 10 | -1.46E-01 | 2.37E-02 | 7.61E-10 | 3.78E+01 |
| CD-European | rs1332099 | C | T | 5.15E-01 | 10 | -2.12E-01 | 2.31E-02 | 4.36E-20 | 8.39E+01 |
| CD-European | rs11236797 | A | C | 4.73E-01 | 11 | 1.81E-01 | 2.31E-02 | 4.85E-15 | 6.15E+01 |
| CD-European | rs11564236 | T | A | 3.44E-02 | 12 | 5.19E-01 | 5.95E-02 | 2.85E-18 | 7.61E+01 |
| CD-European | rs1932990 | T | C | 2.54E-01 | 13 | 1.53E-01 | 2.63E-02 | 6.02E-09 | 3.38E+01 |
| CD-European | rs4902642 | A | G | 4.09E-01 | 14 | -1.29E-01 | 2.36E-02 | 4.34E-08 | 3.00E+01 |
| CD-European | rs56062135 | T | C | 2.34E-01 | 15 | 1.93E-01 | 2.69E-02 | 7.45E-13 | 5.15E+01 |
| CD-European | rs147684209 | C | T | 3.69E-01 | 16 | 1.55E-01 | 2.44E-02 | 2.34E-10 | 4.03E+01 |
| CD-European | rs72798422 | C | T | 4.77E-02 | 16 | 5.90E-01 | 5.08E-02 | 3.19E-31 | 1.35E+02 |
| CD-European | rs2076756 | G | A | 2.84E-01 | 16 | 4.00E-01 | 2.42E-02 | 3.24E-61 | 2.73E+02 |
| CD-European | rs3091315 | G | A | 2.66E-01 | 17 | -1.80E-01 | 2.63E-02 | 9.52E-12 | 4.66E+01 |
| CD-European | rs907092 | A | G | 4.71E-01 | 17 | 1.30E-01 | 2.28E-02 | 1.01E-08 | 3.27E+01 |
| CD-European | rs744166 | G | A | 4.08E-01 | 17 | -1.29E-01 | 2.33E-02 | 2.92E-08 | 3.08E+01 |
| CD-European | rs80262450 | A | G | 1.13E-01 | 18 | 2.83E-01 | 3.53E-02 | 1.08E-15 | 6.43E+01 |
| CD-European | rs8178977 | C | G | 2.39E-01 | 19 | 1.93E-01 | 2.74E-02 | 2.06E-12 | 4.95E+01 |
| CD-European | rs2129944 | G | T | 2.91E-01 | 19 | -1.56E-01 | 2.71E-02 | 7.81E-09 | 3.32E+01 |
| CD-European | rs281379 | A | G | 4.89E-01 | 19 | 1.40E-01 | 2.38E-02 | 4.26E-09 | 3.45E+01 |
| CD-European | rs1056441 | C | T | 6.98E-01 | 20 | 1.67E-01 | 2.55E-02 | 5.44E-11 | 4.29E+01 |
| CD-European | rs1297271 | T | C | 4.30E-01 | 21 | -1.55E-01 | 2.37E-02 | 6.28E-11 | 4.27E+01 |
| CD-European | rs7276302 | G | A | 6.08E-01 | 21 | -1.72E-01 | 2.31E-02 | 1.23E-13 | 5.52E+01 |
| CD-European | rs8137950 | C | T | 2.00E-01 | 22 | 1.74E-01 | 2.86E-02 | 1.17E-09 | 3.70E+01 |
| CD-European | rs151314883 | A | G | 1.58E-01 | 22 | -2.24E-01 | 3.27E-02 | 7.12E-12 | 4.69E+01 |

**Supplementary Table2.** SNPs selected as IVs for CD in European population.

| **exposure** | **SNP** | **effect_allele** | **other_allele** | **eaf** | **chr** | **beta** | **se** | **pval** | **F_statistic** |
| --- | --- | --- | --- | --- | --- | --- | --- | --- | --- |
| IBD-European | rs7523442 | T | C | 5.36E-01 | 1 | 1.25E-01 | 9.90E-03 | 2.76E-36 | 1.58E+02 |
| IBD-European | rs34856868 | A | G | 3.16E-02 | 1 | -1.95E-01 | 3.41E-02 | 9.80E-09 | 3.29E+01 |
| IBD-European | rs2488397 | C | G | 2.04E-01 | 1 | 9.88E-02 | 1.22E-02 | 4.55E-16 | 6.60E+01 |
| IBD-European | rs72634258 | C | T | 1.76E-01 | 1 | -1.27E-01 | 1.40E-02 | 1.25E-19 | 8.22E+01 |
| IBD-European | rs2974935 | T | G | 4.95E-01 | 1 | 6.87E-02 | 1.01E-02 | 8.87E-12 | 4.66E+01 |
| IBD-European | rs12411259 | A | G | 2.40E-01 | 1 | 6.69E-02 | 1.15E-02 | 6.18E-09 | 3.38E+01 |
| IBD-European | rs35730213 | C | G | 2.81E-01 | 1 | -1.60E-01 | 1.14E-02 | 8.33E-45 | 1.97E+02 |
| IBD-European | rs3024493 | A | C | 1.57E-01 | 1 | 1.97E-01 | 1.32E-02 | 1.65E-50 | 2.23E+02 |
| IBD-European | rs12103 | C | T | 8.17E-01 | 1 | -8.67E-02 | 1.31E-02 | 3.28E-11 | 4.40E+01 |
| IBD-European | rs7547569 | C | T | 6.68E-02 | 1 | -6.47E-01 | 2.33E-02 | 1.65E-170 | 7.75E+02 |
| IBD-European | rs2297559 | A | G | 6.82E-01 | 1 | 7.42E-02 | 1.10E-02 | 1.88E-11 | 4.51E+01 |
| IBD-European | rs6588248 | G | T | 5.30E-01 | 1 | 8.20E-02 | 9.92E-03 | 1.38E-16 | 6.83E+01 |
| IBD-European | rs10800309 | G | A | 6.58E-01 | 1 | -1.32E-01 | 1.04E-02 | 6.15E-37 | 1.61E+02 |
| IBD-European | rs13407913 | G | A | 4.31E-01 | 2 | 9.17E-02 | 9.88E-03 | 1.69E-20 | 8.61E+01 |
| IBD-European | rs78487399 | C | G | 9.03E-01 | 2 | -1.32E-01 | 1.64E-02 | 7.71E-16 | 6.49E+01 |
| IBD-European | rs1517352 | C | A | 6.05E-01 | 2 | 7.79E-02 | 1.03E-02 | 3.87E-14 | 5.72E+01 |
| IBD-European | rs6708373 | G | A | 5.28E-01 | 2 | 1.34E-01 | 9.93E-03 | 1.43E-41 | 1.82E+02 |
| IBD-European | rs7608910 | G | A | 3.91E-01 | 2 | 1.26E-01 | 1.00E-02 | 2.60E-36 | 1.58E+02 |
| IBD-European | rs11691685 | G | A | 8.02E-02 | 2 | -1.22E-01 | 1.88E-02 | 7.27E-11 | 4.24E+01 |
| IBD-European | rs6745185 | G | T | 7.39E-01 | 2 | 6.98E-02 | 1.15E-02 | 1.37E-09 | 3.67E+01 |
| IBD-European | rs780094 | C | T | 6.05E-01 | 2 | -7.83E-02 | 9.96E-03 | 3.88E-15 | 6.18E+01 |
| IBD-European | rs1420098 | C | T | 3.88E-01 | 2 | -9.53E-02 | 1.03E-02 | 1.83E-20 | 8.60E+01 |
| IBD-European | rs72924296 | G | A | 2.70E-01 | 2 | -6.38E-02 | 1.13E-02 | 1.44E-08 | 3.21E+01 |
| IBD-European | rs6740462 | A | C | 7.38E-01 | 2 | 8.00E-02 | 1.16E-02 | 5.59E-12 | 4.75E+01 |
| IBD-European | rs1990760 | T | C | 6.09E-01 | 2 | -6.71E-02 | 1.07E-02 | 3.56E-10 | 3.93E+01 |
| IBD-European | rs11677953 | A | G | 3.97E-01 | 2 | 7.91E-02 | 1.00E-02 | 2.92E-15 | 6.23E+01 |
| IBD-European | rs35256947 | C | T | 2.59E-01 | 2 | 8.22E-02 | 1.13E-02 | 3.87E-13 | 5.27E+01 |
| IBD-European | rs9836291 | A | G | 2.88E-01 | 3 | 1.61E-01 | 1.05E-02 | 9.61E-53 | 2.34E+02 |
| IBD-European | rs11713774 | C | T | 1.43E-01 | 3 | 9.43E-02 | 1.43E-02 | 3.92E-11 | 4.37E+01 |
| IBD-European | rs4692386 | C | T | 5.93E-01 | 4 | 5.80E-02 | 1.02E-02 | 1.21E-08 | 3.25E+01 |
| IBD-European | rs7657746 | G | A | 2.44E-01 | 4 | -8.69E-02 | 1.18E-02 | 1.83E-13 | 5.42E+01 |
| IBD-European | rs13107612 | T | C | 2.97E-01 | 4 | 7.33E-02 | 1.09E-02 | 1.62E-11 | 4.54E+01 |
| IBD-European | rs974801 | G | A | 3.80E-01 | 4 | -7.28E-02 | 1.01E-02 | 7.07E-13 | 5.15E+01 |
| IBD-European | rs3776414 | G | T | 3.76E-01 | 5 | 7.74E-02 | 1.02E-02 | 2.65E-14 | 5.80E+01 |
| IBD-European | rs7711427 | C | A | 6.13E-01 | 5 | 1.75E-01 | 1.02E-02 | 4.63E-66 | 2.95E+02 |
| IBD-European | rs4703855 | T | C | 3.00E-01 | 5 | -7.11E-02 | 1.09E-02 | 7.16E-11 | 4.25E+01 |
| IBD-European | rs36048684 | A | T | 1.11E-01 | 5 | -9.41E-02 | 1.60E-02 | 3.70E-09 | 3.48E+01 |
| IBD-European | rs4976646 | C | T | 3.42E-01 | 5 | 7.30E-02 | 1.05E-02 | 3.23E-12 | 4.85E+01 |
| IBD-European | rs79980175 | C | A | 1.36E-01 | 5 | -9.53E-02 | 1.48E-02 | 1.30E-10 | 4.13E+01 |
| IBD-European | rs34804116 | A | C | 3.87E-01 | 5 | -5.75E-02 | 1.04E-02 | 3.62E-08 | 3.03E+01 |
| IBD-European | rs181826 | A | C | 6.27E-01 | 5 | 8.20E-02 | 1.04E-02 | 4.05E-15 | 6.17E+01 |
| IBD-European | rs1363907 | A | G | 4.21E-01 | 5 | 8.15E-02 | 1.04E-02 | 4.87E-15 | 6.13E+01 |
| IBD-European | rs272882 | T | G | 6.73E-01 | 5 | 1.66E-01 | 1.09E-02 | 1.47E-52 | 2.33E+02 |
| IBD-European | rs71593329 | G | T | 2.00E-01 | 5 | -9.78E-02 | 1.27E-02 | 1.19E-14 | 5.96E+01 |
| IBD-European | rs56167332 | A | C | 3.38E-01 | 5 | 1.56E-01 | 1.05E-02 | 7.17E-50 | 2.20E+02 |
| IBD-European | rs6456426 | A | C | 4.98E-01 | 6 | -6.43E-02 | 9.90E-03 | 8.18E-11 | 4.22E+01 |
| IBD-European | rs9264942 | C | T | 3.53E-01 | 6 | 9.47E-02 | 1.08E-02 | 1.55E-18 | 7.72E+01 |
| IBD-European | rs9273363 | A | C | 2.75E-01 | 6 | -1.93E-01 | 1.20E-02 | 3.30E-58 | 2.59E+02 |
| IBD-European | rs1847472 | A | C | 3.42E-01 | 6 | -6.73E-02 | 1.09E-02 | 6.63E-10 | 3.81E+01 |
| IBD-European | rs11758694 | A | T | 9.94E-02 | 6 | 1.08E-01 | 1.60E-02 | 1.75E-11 | 4.52E+01 |
| IBD-European | rs13204742 | T | G | 1.27E-01 | 6 | 9.16E-02 | 1.48E-02 | 5.39E-10 | 3.85E+01 |
| IBD-European | rs1267499 | C | T | 8.10E-01 | 6 | 8.21E-02 | 1.25E-02 | 5.22E-11 | 4.31E+01 |
| IBD-European | rs769177 | T | C | 2.55E-02 | 6 | 2.61E-01 | 2.86E-02 | 6.53E-20 | 8.35E+01 |
| IBD-European | rs6933404 | C | T | 2.11E-01 | 6 | 9.58E-02 | 1.23E-02 | 5.84E-15 | 6.10E+01 |
| IBD-European | rs62434177 | A | G | 3.24E-02 | 6 | -1.79E-01 | 3.14E-02 | 1.14E-08 | 3.26E+01 |
| IBD-European | rs9457247 | T | C | 5.40E-01 | 6 | 8.92E-02 | 1.02E-02 | 2.48E-18 | 7.63E+01 |
| IBD-European | rs7773324 | A | G | 6.00E-01 | 6 | 6.18E-02 | 1.06E-02 | 5.84E-09 | 3.39E+01 |
| IBD-European | rs2328546 | C | T | 8.01E-01 | 6 | 9.40E-02 | 1.27E-02 | 1.30E-13 | 5.49E+01 |
| IBD-European | rs11152949 | G | A | 3.20E-01 | 6 | 1.05E-01 | 1.07E-02 | 7.25E-23 | 9.69E+01 |
| IBD-European | rs3801835 | T | C | 3.45E-01 | 7 | 6.41E-02 | 1.06E-02 | 1.47E-09 | 3.66E+01 |
| IBD-European | rs11768997 | T | G | 1.35E-01 | 7 | 1.55E-01 | 1.80E-02 | 6.82E-18 | 7.43E+01 |
| IBD-European | rs6466198 | T | A | 3.86E-01 | 7 | 8.41E-02 | 1.02E-02 | 2.18E-16 | 6.74E+01 |
| IBD-European | rs2538470 | G | A | 6.38E-01 | 7 | -6.76E-02 | 1.02E-02 | 3.00E-11 | 4.42E+01 |
| IBD-European | rs1182188 | C | T | 2.99E-01 | 7 | -6.59E-02 | 1.08E-02 | 1.08E-09 | 3.72E+01 |
| IBD-European | rs12718244 | A | G | 4.08E-01 | 7 | 7.62E-02 | 1.00E-02 | 3.35E-14 | 5.75E+01 |
| IBD-European | rs2395022 | C | A | 9.59E-01 | 7 | -1.82E-01 | 2.34E-02 | 8.27E-15 | 6.03E+01 |
| IBD-European | rs7011507 | A | G | 1.23E-01 | 8 | -8.46E-02 | 1.51E-02 | 2.03E-08 | 3.15E+01 |
| IBD-European | rs7015630 | C | T | 2.66E-01 | 8 | -6.28E-02 | 1.13E-02 | 2.90E-08 | 3.08E+01 |
| IBD-European | rs10956252 | G | C | 6.19E-01 | 8 | 8.35E-02 | 1.02E-02 | 2.26E-16 | 6.74E+01 |
| IBD-European | rs6651252 | C | T | 1.30E-01 | 8 | -9.08E-02 | 1.48E-02 | 9.08E-10 | 3.75E+01 |
| IBD-European | rs10758669 | A | C | 6.50E-01 | 9 | -1.49E-01 | 1.02E-02 | 4.70E-48 | 2.12E+02 |
| IBD-European | rs7848647 | C | T | 6.75E-01 | 9 | 1.32E-01 | 1.07E-02 | 3.16E-35 | 1.53E+02 |
| IBD-European | rs4743820 | T | C | 7.02E-01 | 9 | 6.40E-02 | 1.09E-02 | 3.80E-09 | 3.47E+01 |
| IBD-European | rs11793497 | G | A | 4.23E-01 | 9 | 1.56E-01 | 1.00E-02 | 1.71E-54 | 2.42E+02 |
| IBD-European | rs34779708 | G | T | 3.51E-01 | 10 | 1.07E-01 | 1.02E-02 | 2.07E-25 | 1.09E+02 |
| IBD-European | rs11185982 | C | T | 1.52E-01 | 10 | -7.79E-02 | 1.38E-02 | 1.61E-08 | 3.19E+01 |
| IBD-European | rs2497318 | T | C | 4.50E-01 | 10 | -6.35E-02 | 9.90E-03 | 1.36E-10 | 4.12E+01 |
| IBD-European | rs2050392 | A | G | 6.00E-01 | 10 | 6.91E-02 | 1.03E-02 | 1.87E-11 | 4.51E+01 |
| IBD-European | rs1250566 | A | G | 3.16E-01 | 10 | -1.01E-01 | 1.10E-02 | 4.77E-20 | 8.41E+01 |
| IBD-European | rs2274351 | T | C | 5.37E-01 | 10 | 6.05E-02 | 1.04E-02 | 6.93E-09 | 3.36E+01 |
| IBD-European | rs12722515 | A | C | 1.63E-01 | 10 | -9.89E-02 | 1.43E-02 | 4.57E-12 | 4.79E+01 |
| IBD-European | rs2153283 | A | C | 2.17E-01 | 10 | -8.60E-02 | 1.27E-02 | 1.54E-11 | 4.55E+01 |
| IBD-European | rs10761659 | G | A | 5.40E-01 | 10 | 1.54E-01 | 1.00E-02 | 4.97E-53 | 2.35E+02 |
| IBD-European | rs2688608 | T | G | 5.57E-01 | 10 | 6.24E-02 | 9.89E-03 | 2.75E-10 | 3.98E+01 |
| IBD-European | rs6584281 | G | A | 5.19E-01 | 10 | -1.65E-01 | 9.93E-03 | 9.36E-62 | 2.75E+02 |
| IBD-European | rs12796489 | A | C | 2.29E-02 | 11 | -7.60E-01 | 4.32E-02 | 2.87E-69 | 3.09E+02 |
| IBD-European | rs11230563 | T | C | 3.48E-01 | 11 | -8.12E-02 | 1.06E-02 | 1.71E-14 | 5.88E+01 |
| IBD-European | rs11236797 | A | C | 4.44E-01 | 11 | 1.51E-01 | 9.97E-03 | 9.32E-52 | 2.29E+02 |
| IBD-European | rs559928 | C | T | 8.13E-01 | 11 | 9.44E-02 | 1.30E-02 | 3.33E-13 | 5.30E+01 |
| IBD-European | rs648541 | G | A | 3.41E-01 | 11 | -6.49E-02 | 1.07E-02 | 1.22E-09 | 3.69E+01 |
| IBD-European | rs10878302 | A | T | 9.29E-01 | 12 | 1.12E-01 | 1.93E-02 | 5.26E-09 | 3.41E+01 |
| IBD-European | rs1388585 | A | G | 9.81E-01 | 12 | -3.05E-01 | 3.17E-02 | 6.85E-22 | 9.25E+01 |
| IBD-European | rs12318183 | A | C | 3.85E-01 | 12 | 1.10E-01 | 1.01E-02 | 1.67E-27 | 1.18E+02 |
| IBD-European | rs3184504 | C | T | 5.07E-01 | 12 | -6.00E-02 | 9.89E-03 | 1.29E-09 | 3.68E+01 |
| IBD-European | rs941823 | C | T | 7.51E-01 | 13 | 8.30E-02 | 1.15E-02 | 6.19E-13 | 5.18E+01 |
| IBD-European | rs9557207 | G | A | 2.23E-01 | 13 | -8.78E-02 | 1.21E-02 | 3.52E-13 | 5.29E+01 |
| IBD-European | rs12585310 | A | G | 3.14E-01 | 13 | 7.06E-02 | 1.08E-02 | 5.25E-11 | 4.31E+01 |
| IBD-European | rs6561151 | A | G | 2.24E-01 | 13 | 1.00E-01 | 1.19E-02 | 3.53E-17 | 7.10E+01 |
| IBD-European | rs1569328 | T | C | 1.70E-01 | 14 | -8.10E-02 | 1.37E-02 | 3.21E-09 | 3.51E+01 |
| IBD-European | rs10142466 | G | A | 5.07E-01 | 14 | -5.80E-02 | 1.01E-02 | 1.08E-08 | 3.27E+01 |
| IBD-European | rs55808324 | A | G | 9.32E-02 | 14 | 1.41E-01 | 1.68E-02 | 5.13E-17 | 7.03E+01 |
| IBD-European | rs17293632 | T | C | 2.36E-01 | 15 | 1.07E-01 | 1.16E-02 | 2.71E-20 | 8.52E+01 |
| IBD-European | rs17651741 | A | G | 1.91E-01 | 15 | 7.02E-02 | 1.27E-02 | 2.81E-08 | 3.08E+01 |
| IBD-European | rs6500315 | G | A | 7.75E-01 | 16 | 7.66E-02 | 1.19E-02 | 1.12E-10 | 4.16E+01 |
| IBD-European | rs62037363 | C | T | 3.91E-01 | 16 | 9.88E-02 | 1.03E-02 | 6.36E-22 | 9.26E+01 |
| IBD-European | rs367569 | T | C | 2.89E-01 | 16 | -9.58E-02 | 1.13E-02 | 1.93E-17 | 7.22E+01 |
| IBD-European | rs7194886 | T | C | 4.36E-01 | 16 | -1.26E-01 | 1.00E-02 | 2.53E-36 | 1.58E+02 |
| IBD-European | rs2270395 | T | C | 7.62E-01 | 16 | 7.78E-02 | 1.19E-02 | 5.17E-11 | 4.31E+01 |
| IBD-European | rs11641016 | G | C | 1.97E-01 | 16 | -1.11E-01 | 1.34E-02 | 9.51E-17 | 6.91E+01 |
| IBD-European | rs3853824 | C | T | 6.39E-01 | 17 | 6.40E-02 | 1.04E-02 | 7.70E-10 | 3.78E+01 |
| IBD-European | rs4795397 | G | A | 4.71E-01 | 17 | 1.38E-01 | 9.97E-03 | 8.30E-44 | 1.93E+02 |
| IBD-European | rs744166 | G | A | 4.20E-01 | 17 | -1.00E-01 | 1.02E-02 | 1.14E-22 | 9.60E+01 |
| IBD-European | rs9889296 | A | G | 2.72E-01 | 17 | -1.05E-01 | 1.13E-02 | 1.35E-20 | 8.66E+01 |
| IBD-European | rs1292053 | G | A | 4.42E-01 | 17 | 7.01E-02 | 9.83E-03 | 9.89E-13 | 5.09E+01 |
| IBD-European | rs17780256 | C | A | 1.93E-01 | 17 | -8.34E-02 | 1.26E-02 | 3.19E-11 | 4.41E+01 |
| IBD-European | rs7240004 | G | A | 3.80E-01 | 18 | -6.65E-02 | 1.03E-02 | 1.01E-10 | 4.18E+01 |
| IBD-European | rs67643815 | T | G | 5.34E-01 | 18 | -6.29E-02 | 1.02E-02 | 6.42E-10 | 3.82E+01 |
| IBD-European | rs2847278 | T | C | 8.41E-01 | 18 | -1.45E-01 | 1.32E-02 | 8.33E-28 | 1.19E+02 |
| IBD-European | rs35164067 | A | G | 2.04E-01 | 19 | -1.18E-01 | 1.27E-02 | 2.66E-20 | 8.52E+01 |
| IBD-European | rs7253253 | T | G | 9.54E-01 | 19 | -1.34E-01 | 2.31E-02 | 6.19E-09 | 3.38E+01 |
| IBD-European | rs2024092 | A | G | 2.16E-01 | 19 | 1.07E-01 | 1.21E-02 | 1.12E-18 | 7.78E+01 |
| IBD-European | rs17694108 | A | G | 2.80E-01 | 19 | 8.58E-02 | 1.11E-02 | 1.21E-14 | 5.95E+01 |
| IBD-European | rs516246 | T | C | 4.65E-01 | 19 | 7.56E-02 | 1.02E-02 | 1.15E-13 | 5.51E+01 |
| IBD-European | rs6111031 | T | C | 1.59E-01 | 20 | -2.64E-01 | 1.48E-02 | 1.23E-71 | 3.20E+02 |
| IBD-European | rs913678 | C | T | 3.29E-01 | 20 | -6.92E-02 | 1.05E-02 | 5.35E-11 | 4.30E+01 |
| IBD-European | rs6058869 | T | C | 3.99E-01 | 20 | 5.57E-02 | 1.00E-02 | 2.63E-08 | 3.10E+01 |
| IBD-European | rs6074022 | T | C | 7.50E-01 | 20 | -7.43E-02 | 1.14E-02 | 8.32E-11 | 4.22E+01 |
| IBD-European | rs6062496 | A | G | 5.69E-01 | 20 | 1.23E-01 | 1.02E-02 | 2.11E-33 | 1.45E+02 |
| IBD-European | rs259964 | G | A | 5.41E-01 | 20 | -6.75E-02 | 9.83E-03 | 6.93E-12 | 4.70E+01 |
| IBD-European | rs2836883 | A | G | 2.73E-01 | 21 | -1.68E-01 | 1.15E-02 | 3.38E-48 | 2.13E+02 |
| IBD-European | rs1297258 | T | C | 4.25E-01 | 21 | -1.15E-01 | 1.01E-02 | 5.38E-30 | 1.29E+02 |
| IBD-European | rs8127691 | C | T | 6.13E-01 | 21 | -1.14E-01 | 1.01E-02 | 8.98E-30 | 1.28E+02 |
| IBD-European | rs140143 | T | G | 3.90E-01 | 22 | -1.33E-01 | 1.14E-02 | 2.18E-31 | 1.36E+02 |
| IBD-European | rs2143178 | C | T | 1.66E-01 | 22 | -1.77E-01 | 1.37E-02 | 4.80E-38 | 1.66E+02 |

**Supplementary Table3.** SNPs selected as IVs for overall IBD in European population.

| **exposure** | **SNP** | **effect_allele** | **other_allele** | **eaf** | **chr** | **beta** | **se** | **pval** | **F_statistic** |
| --- | --- | --- | --- | --- | --- | --- | --- | --- | --- |
| UC-East Asian | rs10465507 | A | C | 4.97E-02 | 1 | -7.99E-01 | 1.40E-01 | 9.87E-09 | 3.27E+01 |
| UC-East Asian | rs13397737 | T | G | 4.36E-02 | 2 | -1.11E+00 | 1.76E-01 | 2.76E-10 | 3.96E+01 |
| UC-East Asian | rs2844623 | T | C | 2.18E-01 | 6 | 5.09E-01 | 5.23E-02 | 1.35E-22 | 9.46E+01 |
| UC-East Asian | rs13203429 | C | G | 5.22E-01 | 6 | 3.46E-01 | 4.84E-02 | 7.01E-13 | 5.12E+01 |
| UC-East Asian | rs7741807 | C | G | 1.81E-01 | 6 | 5.49E-01 | 5.48E-02 | 7.57E-24 | 1.00E+02 |
| UC-East Asian | rs2647015 | G | T | 2.05E-01 | 6 | 6.21E-01 | 5.24E-02 | 7.09E-33 | 1.40E+02 |
| UC-East Asian | rs2274200 | C | T | 2.52E-01 | 6 | 2.93E-01 | 5.20E-02 | 1.60E-08 | 3.18E+01 |
| UC-East Asian | rs79315643 | G | A | 9.47E-02 | 8 | -5.86E-01 | 9.63E-02 | 1.06E-09 | 3.70E+01 |
| UC-East Asian | rs10975003 | C | T | 1.89E-01 | 9 | 3.23E-01 | 5.57E-02 | 6.08E-09 | 3.37E+01 |
| UC-East Asian | rs2940716 | A | G | 1.35E-04 | 10 | 3.12E+00 | 5.00E-01 | 4.11E-10 | 3.89E+01 |

**Supplementary Table4.** SNPs selected as IVs for UC in East Asian population.

| **exposure** | **SNP** | **effect_allele** | **other_allele** | **eaf** | **chr** | **beta** | **se** | **pval** | **F_statistic** |
| --- | --- | --- | --- | --- | --- | --- | --- | --- | --- |
| CD-East Asian | rs2975785 | G | A | 7.30E-01 | 2 | -2.45E-01 | 4.48E-02 | 4.63E-08 | 2.99E+01 |
| CD-East Asian | rs6856616 | C | T | 2.20E-01 | 4 | 2.68E-01 | 4.75E-02 | 1.76E-08 | 3.17E+01 |
| CD-East Asian | rs60689680 | T | G | 4.10E-01 | 5 | 2.71E-01 | 4.19E-02 | 9.62E-11 | 4.19E+01 |
| CD-East Asian | rs9368758 | A | G | 5.32E-01 | 6 | 2.35E-01 | 4.15E-02 | 1.44E-08 | 3.21E+01 |
| CD-East Asian | rs4713586 | G | A | 1.55E-01 | 6 | 6.53E-01 | 4.97E-02 | 2.07E-39 | 1.72E+02 |
| CD-East Asian | rs6930777 | T | C | 4.84E-02 | 6 | -9.65E-01 | 1.32E-01 | 2.67E-13 | 5.34E+01 |
| CD-East Asian | rs2395449 | A | T | 7.01E-01 | 6 | 3.09E-01 | 4.86E-02 | 2.01E-10 | 4.05E+01 |
| CD-East Asian | rs36110421 | C | A | 8.95E-02 | 6 | -5.18E-01 | 8.61E-02 | 1.81E-09 | 3.62E+01 |
| CD-East Asian | rs16894919 | T | G | 1.88E-01 | 6 | -3.29E-01 | 5.69E-02 | 7.46E-09 | 3.34E+01 |
| CD-East Asian | rs79315643 | G | A | 9.47E-02 | 8 | -8.26E-01 | 8.90E-02 | 1.52E-20 | 8.62E+01 |
| CD-East Asian | rs4366152 | C | T | 6.01E-01 | 9 | 6.42E-01 | 4.55E-02 | 2.52E-45 | 1.99E+02 |
| CD-East Asian | rs2006996 | C | T | 5.00E-01 | 9 | 5.24E-01 | 4.28E-02 | 1.74E-34 | 1.50E+02 |
| CD-East Asian | rs224090 | C | T | 5.20E-01 | 10 | -2.47E-01 | 4.17E-02 | 3.03E-09 | 3.52E+01 |
| CD-East Asian | rs10748783 | A | C | 5.87E-01 | 10 | -2.81E-01 | 4.30E-02 | 6.36E-11 | 4.27E+01 |

**Supplementary Table5.** SNPs selected as IVs for CD in East Asian population.

| **exposure** | **SNP** | **effect_allele** | **other_allele** | **eaf** | **chr** | **beta** | **se** | **pval** | **F_statistic** |
| --- | --- | --- | --- | --- | --- | --- | --- | --- | --- |
| IBD-East Asian | rs4113832 | T | C | 5.69E-02 | 1 | -4.79E-01 | 8.47E-02 | 1.65E-08 | 3.19E+01 |
| IBD-East Asian | rs13397737 | T | G | 4.36E-02 | 2 | -7.88E-01 | 1.11E-01 | 1.13E-12 | 5.08E+01 |
| IBD-East Asian | rs56167332 | A | C | 3.72E-01 | 5 | 2.13E-01 | 3.61E-02 | 3.70E-09 | 3.49E+01 |
| IBD-East Asian | rs7775228 | C | T | 3.60E-01 | 6 | 3.74E-01 | 3.57E-02 | 1.78E-25 | 1.10E+02 |
| IBD-East Asian | rs2621383 | A | C | 7.67E-01 | 6 | 2.34E-01 | 4.26E-02 | 4.26E-08 | 3.01E+01 |
| IBD-East Asian | rs422544 | C | A | 3.34E-01 | 6 | -2.62E-01 | 3.83E-02 | 8.39E-12 | 4.68E+01 |
| IBD-East Asian | rs79315643 | G | A | 9.47E-02 | 8 | -7.45E-01 | 7.09E-02 | 1.20E-25 | 1.10E+02 |
| IBD-East Asian | rs77725210 | G | A | 4.08E-01 | 9 | 2.24E-01 | 3.58E-02 | 4.36E-10 | 3.90E+01 |
| IBD-East Asian | rs6478108 | T | C | 5.97E-01 | 9 | 4.22E-01 | 3.65E-02 | 1.11E-30 | 1.34E+02 |
| IBD-East Asian | rs224090 | C | T | 5.20E-01 | 10 | -2.05E-01 | 3.57E-02 | 1.00E-08 | 3.29E+01 |
| IBD-East Asian | rs10748783 | A | C | 5.87E-01 | 10 | -2.52E-01 | 3.61E-02 | 3.47E-12 | 4.86E+01 |

**Supplementary Table6.** SNPs selected as IVs for overall IBD in East Asian population.

|  |  |  | **pleiotropy_test** | **heterogeneity_test** | | | | | |
| --- | --- | --- | --- | --- | --- | --- | --- | --- | --- |
| **exposure** | **outcome** | **ethnic** | **p_value** | **method** | **Q statistic** | **p_value** | **method** | **Q statistic** | **p_value** |
| UC | BMD | European | 8.91E-01 | MR-Egger | 1.66E+02 | 1.74E-07 | IVW | 1.66E+02 | 2.47E-07 |
| CD | BMD | European | 1.81E-01 | MR-Egger | 7.60E+01 | 8.00E-03 | IVW | 7.88E+01 | 5.70E-03 |
| IBD | BMD | European | 1.74E-01 | MR-Egger | 2.43E+02 | 5.82E-09 | IVW | 2.46E+02 | 3.44E-09 |
| UC | OS with FG | European | 9.90E-02 | MR-Egger | 8.26E+01 | 4.00E-01 | IVW | 8.55E+01 | 3.46E-01 |
| CD | OS with FG | European | 9.53E-01 | MR-Egger | 4.28E+01 | 6.06E-01 | IVW | 4.28E+01 | 6.45E-01 |
| IBD | OS with FG | European | 6.25E-01 | MR-Egger | 1.21E+02 | 6.24E-01 | IVW | 1.22E+02 | 6.42E-01 |
| UC | OS | East Asian | 7.16E-01 | MR-Egger | 1.36E+01 | 1.80E-02 | IVW | 1.40E+01 | 2.91E-02 |
| CD | OS | East Asian | 4.02E-01 | MR-Egger | 1.79E+01 | 1.17E-01 | IVW | 1.91E+01 | 1.21E-01 |
| IBD | OS | East Asian | 9.03E-01 | MR-Egger | 1.23E+01 | 1.37E-01 | IVW | 1.24E+01 | 1.94E-01 |

**Supplementary Table7.** Pleiotropy and heterogeneity test results of all two-sample MR analyses.

| **SNP** | **effect allele** | **other allele** | **se** | **beta** | **OR** | **lo_CI** | **up_CI** | **pval** | **eaf** | **chr** |
| --- | --- | --- | --- | --- | --- | --- | --- | --- | --- | --- |
| rs780094 | C | T | 5.80E-03 | 3.01E-02 | 1.03E+00 | 1.02E+00 | 1.04E+00 | 2.54E-07 | 6.23E-01 | 2 |
| rs516246 | T | C | 5.90E-03 | -2.60E-02 | 9.74E-01 | 9.63E-01 | 9.86E-01 | 1.17E-05 | 4.84E-01 | 19 |
| rs3184504 | C | T | 5.80E-03 | 1.93E-02 | 1.02E+00 | 1.01E+00 | 1.03E+00 | 8.45E-04 | 5.63E-01 | 12 |
| rs6708373 | G | A | 5.90E-03 | -1.91E-02 | 9.81E-01 | 9.70E-01 | 9.92E-01 | 1.22E-03 | 5.10E-01 | 2 |
| rs11152949 | G | A | 6.20E-03 | 1.99E-02 | 1.02E+00 | 1.01E+00 | 1.03E+00 | 1.36E-03 | 2.93E-01 | 6 |
| rs6456426 | A | C | 5.90E-03 | 1.76E-02 | 1.02E+00 | 1.01E+00 | 1.03E+00 | 2.77E-03 | 4.79E-01 | 6 |
| rs9836291 | A | G | 6.20E-03 | -1.78E-02 | 9.82E-01 | 9.70E-01 | 9.94E-01 | 4.35E-03 | 2.90E-01 | 3 |
| rs9273363 | A | C | 7.80E-03 | 2.03E-02 | 1.02E+00 | 1.01E+00 | 1.04E+00 | 8.72E-03 | 2.80E-01 | 6 |
| rs6111031 | T | C | 8.40E-03 | -2.18E-02 | 9.78E-01 | 9.62E-01 | 9.95E-01 | 9.29E-03 | 1.36E-01 | 20 |
| rs4703855 | T | C | 6.20E-03 | 1.54E-02 | 1.02E+00 | 1.00E+00 | 1.03E+00 | 1.31E-02 | 2.94E-01 | 5 |
| rs4743820 | T | C | 6.20E-03 | -1.52E-02 | 9.85E-01 | 9.73E-01 | 9.97E-01 | 1.47E-02 | 6.90E-01 | 9 |
| rs78487399 | C | G | 1.00E-02 | -2.29E-02 | 9.77E-01 | 9.58E-01 | 9.97E-01 | 2.21E-02 | 9.11E-01 | 2 |
| rs72924296 | G | A | 6.70E-03 | 1.44E-02 | 1.01E+00 | 1.00E+00 | 1.03E+00 | 3.05E-02 | 2.57E-01 | 2 |
| rs10758669 | A | C | 6.10E-03 | 1.29E-02 | 1.01E+00 | 1.00E+00 | 1.03E+00 | 3.30E-02 | 6.60E-01 | 9 |
| rs2847278 | T | C | 7.70E-03 | 1.61E-02 | 1.02E+00 | 1.00E+00 | 1.03E+00 | 3.60E-02 | 8.34E-01 | 18 |
| rs1182188 | C | T | 6.30E-03 | 1.31E-02 | 1.01E+00 | 1.00E+00 | 1.03E+00 | 3.66E-02 | 2.83E-01 | 7 |
| rs62434177 | A | G | 1.83E-02 | -3.78E-02 | 9.63E-01 | 9.29E-01 | 9.98E-01 | 3.85E-02 | 3.39E-02 | 6 |
| rs6074022 | T | C | 6.50E-03 | 1.30E-02 | 1.01E+00 | 1.00E+00 | 1.03E+00 | 4.75E-02 | 7.52E-01 | 20 |
| rs7253253 | T | G | 1.47E-02 | 2.87E-02 | 1.03E+00 | 1.00E+00 | 1.06E+00 | 5.11E-02 | 9.54E-01 | 19 |
| rs17651741 | A | G | 7.20E-03 | -1.39E-02 | 9.86E-01 | 9.72E-01 | 1.00E+00 | 5.46E-02 | 1.81E-01 | 15 |
| rs67643815 | T | G | 5.70E-03 | -1.09E-02 | 9.89E-01 | 9.78E-01 | 1.00E+00 | 5.65E-02 | 5.24E-01 | 18 |
| rs744166 | G | A | 5.70E-03 | 1.05E-02 | 1.01E+00 | 9.99E-01 | 1.02E+00 | 6.49E-02 | 4.43E-01 | 17 |
| rs7240004 | G | A | 5.90E-03 | -1.09E-02 | 9.89E-01 | 9.78E-01 | 1.00E+00 | 6.60E-02 | 3.90E-01 | 18 |
| rs12103 | C | T | 8.10E-03 | 1.47E-02 | 1.01E+00 | 9.99E-01 | 1.03E+00 | 6.85E-02 | 7.33E-01 | 1 |
| rs17293632 | T | C | 6.80E-03 | 1.20E-02 | 1.01E+00 | 9.99E-01 | 1.03E+00 | 7.62E-02 | 2.25E-01 | 15 |
| rs12722515 | A | C | 7.70E-03 | -1.33E-02 | 9.87E-01 | 9.72E-01 | 1.00E+00 | 8.27E-02 | 1.62E-01 | 10 |
| rs7015630 | C | T | 6.50E-03 | -1.13E-02 | 9.89E-01 | 9.76E-01 | 1.00E+00 | 8.31E-02 | 2.67E-01 | 8 |
| rs71593329 | G | T | 7.20E-03 | 1.24E-02 | 1.01E+00 | 9.98E-01 | 1.03E+00 | 8.38E-02 | 1.91E-01 | 5 |
| rs2497318 | T | C | 5.80E-03 | -9.80E-03 | 9.90E-01 | 9.79E-01 | 1.00E+00 | 8.85E-02 | 4.24E-01 | 10 |
| rs7011507 | A | G | 8.90E-03 | -1.50E-02 | 9.85E-01 | 9.68E-01 | 1.00E+00 | 9.21E-02 | 1.19E-01 | 8 |
| rs4692386 | C | T | 6.20E-03 | -1.04E-02 | 9.90E-01 | 9.78E-01 | 1.00E+00 | 9.23E-02 | 5.94E-01 | 4 |
| rs17694108 | A | G | 6.80E-03 | -1.09E-02 | 9.89E-01 | 9.76E-01 | 1.00E+00 | 1.11E-01 | 2.77E-01 | 19 |
| rs272882 | T | G | 6.20E-03 | -9.90E-03 | 9.90E-01 | 9.78E-01 | 1.00E+00 | 1.14E-01 | 7.01E-01 | 5 |
| rs34779708 | G | T | 6.00E-03 | -9.10E-03 | 9.91E-01 | 9.79E-01 | 1.00E+00 | 1.26E-01 | 3.49E-01 | 10 |
| rs7848647 | C | T | 6.10E-03 | -9.30E-03 | 9.91E-01 | 9.79E-01 | 1.00E+00 | 1.26E-01 | 6.87E-01 | 9 |
| rs367569 | T | C | 6.30E-03 | -9.60E-03 | 9.90E-01 | 9.78E-01 | 1.00E+00 | 1.29E-01 | 2.84E-01 | 16 |
| rs72634258 | C | T | 7.70E-03 | 1.13E-02 | 1.01E+00 | 9.96E-01 | 1.03E+00 | 1.43E-01 | 1.72E-01 | 1 |
| rs2297559 | A | G | 6.10E-03 | 8.90E-03 | 1.01E+00 | 9.97E-01 | 1.02E+00 | 1.44E-01 | 6.76E-01 | 1 |
| rs259964 | G | A | 5.70E-03 | 8.20E-03 | 1.01E+00 | 9.97E-01 | 1.02E+00 | 1.48E-01 | 5.49E-01 | 20 |
| rs12718244 | A | G | 5.80E-03 | -8.20E-03 | 9.92E-01 | 9.81E-01 | 1.00E+00 | 1.58E-01 | 3.94E-01 | 7 |
| rs10142466 | G | A | 5.70E-03 | -7.60E-03 | 9.92E-01 | 9.81E-01 | 1.00E+00 | 1.83E-01 | 4.99E-01 | 14 |
| rs35256947 | C | T | 6.60E-03 | 8.70E-03 | 1.01E+00 | 9.96E-01 | 1.02E+00 | 1.90E-01 | 2.42E-01 | 2 |
| rs3801835 | T | C | 6.00E-03 | -7.80E-03 | 9.92E-01 | 9.81E-01 | 1.00E+00 | 1.94E-01 | 3.30E-01 | 7 |
| rs2836883 | A | G | 6.40E-03 | 8.10E-03 | 1.01E+00 | 9.96E-01 | 1.02E+00 | 2.07E-01 | 2.72E-01 | 21 |
| rs2395022 | C | A | 1.39E-02 | 1.75E-02 | 1.02E+00 | 9.90E-01 | 1.05E+00 | 2.09E-01 | 9.48E-01 | 7 |
| rs7657746 | G | A | 6.50E-03 | 8.10E-03 | 1.01E+00 | 9.95E-01 | 1.02E+00 | 2.11E-01 | 2.53E-01 | 4 |
| rs2974935 | T | G | 6.00E-03 | -7.50E-03 | 9.93E-01 | 9.81E-01 | 1.00E+00 | 2.13E-01 | 5.24E-01 | 1 |
| rs11641016 | G | C | 7.50E-03 | -9.30E-03 | 9.91E-01 | 9.76E-01 | 1.01E+00 | 2.14E-01 | 1.98E-01 | 16 |
| rs913678 | C | T | 6.20E-03 | -7.60E-03 | 9.92E-01 | 9.80E-01 | 1.00E+00 | 2.18E-01 | 3.70E-01 | 20 |
| rs648541 | G | A | 6.00E-03 | 7.20E-03 | 1.01E+00 | 9.95E-01 | 1.02E+00 | 2.27E-01 | 3.44E-01 | 11 |
| rs6933404 | C | T | 7.10E-03 | -8.40E-03 | 9.92E-01 | 9.78E-01 | 1.01E+00 | 2.37E-01 | 1.95E-01 | 6 |
| rs6058869 | T | C | 5.80E-03 | -6.70E-03 | 9.93E-01 | 9.82E-01 | 1.00E+00 | 2.52E-01 | 4.39E-01 | 20 |
| rs36048684 | A | T | 9.10E-03 | -9.90E-03 | 9.90E-01 | 9.73E-01 | 1.01E+00 | 2.75E-01 | 1.11E-01 | 5 |
| rs8127691 | C | T | 5.80E-03 | -5.90E-03 | 9.94E-01 | 9.83E-01 | 1.01E+00 | 3.10E-01 | 6.12E-01 | 21 |
| rs1569328 | T | C | 7.80E-03 | -7.90E-03 | 9.92E-01 | 9.77E-01 | 1.01E+00 | 3.13E-01 | 1.60E-01 | 14 |
| rs12585310 | A | G | 6.20E-03 | 6.10E-03 | 1.01E+00 | 9.94E-01 | 1.02E+00 | 3.24E-01 | 3.04E-01 | 13 |
| rs12796489 | A | C | 4.85E-02 | 4.76E-02 | 1.05E+00 | 9.54E-01 | 1.15E+00 | 3.27E-01 | 8.10E-03 | 11 |
| rs181826 | A | C | 5.90E-03 | 5.70E-03 | 1.01E+00 | 9.94E-01 | 1.02E+00 | 3.32E-01 | 6.34E-01 | 5 |
| rs4976646 | C | T | 6.20E-03 | -5.80E-03 | 9.94E-01 | 9.82E-01 | 1.01E+00 | 3.47E-01 | 3.52E-01 | 5 |
| rs1267499 | C | T | 7.30E-03 | -6.80E-03 | 9.93E-01 | 9.79E-01 | 1.01E+00 | 3.56E-01 | 7.60E-01 | 6 |
| rs2143178 | C | T | 7.60E-03 | 6.90E-03 | 1.01E+00 | 9.92E-01 | 1.02E+00 | 3.65E-01 | 1.72E-01 | 22 |
| rs1990760 | T | C | 5.80E-03 | 5.10E-03 | 1.01E+00 | 9.94E-01 | 1.02E+00 | 3.74E-01 | 5.70E-01 | 2 |
| rs12318183 | A | C | 5.80E-03 | 5.20E-03 | 1.01E+00 | 9.94E-01 | 1.02E+00 | 3.76E-01 | 3.72E-01 | 12 |
| rs7711427 | C | A | 5.80E-03 | 4.60E-03 | 1.00E+00 | 9.93E-01 | 1.02E+00 | 4.29E-01 | 5.94E-01 | 5 |
| rs1388585 | A | G | 1.85E-02 | -1.46E-02 | 9.86E-01 | 9.50E-01 | 1.02E+00 | 4.31E-01 | 9.68E-01 | 12 |
| rs6745185 | G | T | 6.50E-03 | -5.10E-03 | 9.95E-01 | 9.82E-01 | 1.01E+00 | 4.38E-01 | 7.44E-01 | 2 |
| rs6561151 | A | G | 6.80E-03 | 5.30E-03 | 1.01E+00 | 9.92E-01 | 1.02E+00 | 4.39E-01 | 2.31E-01 | 13 |
| rs2270395 | T | C | 6.90E-03 | 5.30E-03 | 1.01E+00 | 9.92E-01 | 1.02E+00 | 4.42E-01 | 7.58E-01 | 16 |
| rs6651252 | C | T | 8.30E-03 | 6.10E-03 | 1.01E+00 | 9.90E-01 | 1.02E+00 | 4.63E-01 | 1.43E-01 | 8 |
| rs2050392 | A | G | 6.00E-03 | -4.30E-03 | 9.96E-01 | 9.84E-01 | 1.01E+00 | 4.69E-01 | 5.64E-01 | 10 |
| rs6062496 | A | G | 6.10E-03 | -4.40E-03 | 9.96E-01 | 9.84E-01 | 1.01E+00 | 4.71E-01 | 5.32E-01 | 20 |
| rs34804116 | A | C | 5.80E-03 | 4.00E-03 | 1.00E+00 | 9.93E-01 | 1.02E+00 | 4.83E-01 | 3.93E-01 | 5 |
| rs56167332 | A | C | 6.40E-03 | -4.40E-03 | 9.96E-01 | 9.83E-01 | 1.01E+00 | 4.89E-01 | 3.31E-01 | 5 |
| rs3024493 | A | C | 7.90E-03 | -5.40E-03 | 9.95E-01 | 9.79E-01 | 1.01E+00 | 4.92E-01 | 1.49E-01 | 1 |
| rs12411259 | A | G | 6.50E-03 | -4.50E-03 | 9.96E-01 | 9.83E-01 | 1.01E+00 | 4.93E-01 | 2.67E-01 | 1 |
| rs2488397 | C | G | 7.00E-03 | 4.60E-03 | 1.00E+00 | 9.91E-01 | 1.02E+00 | 5.07E-01 | 2.18E-01 | 1 |
| rs35730213 | C | G | 6.40E-03 | -4.10E-03 | 9.96E-01 | 9.83E-01 | 1.01E+00 | 5.16E-01 | 2.72E-01 | 1 |
| rs6466198 | T | A | 6.00E-03 | 3.90E-03 | 1.00E+00 | 9.92E-01 | 1.02E+00 | 5.21E-01 | 3.82E-01 | 7 |
| rs2538470 | G | A | 5.80E-03 | -3.70E-03 | 9.96E-01 | 9.85E-01 | 1.01E+00 | 5.21E-01 | 6.33E-01 | 7 |
| rs11236797 | A | C | 5.80E-03 | 3.70E-03 | 1.00E+00 | 9.92E-01 | 1.02E+00 | 5.26E-01 | 4.28E-01 | 11 |
| rs7194886 | T | C | 5.80E-03 | 3.70E-03 | 1.00E+00 | 9.92E-01 | 1.02E+00 | 5.26E-01 | 4.04E-01 | 16 |
| rs3776414 | G | T | 5.80E-03 | 3.50E-03 | 1.00E+00 | 9.92E-01 | 1.01E+00 | 5.50E-01 | 4.01E-01 | 5 |
| rs7608910 | G | A | 5.80E-03 | -3.30E-03 | 9.97E-01 | 9.85E-01 | 1.01E+00 | 5.75E-01 | 3.72E-01 | 2 |
| rs769177 | T | C | 1.96E-02 | -1.09E-02 | 9.89E-01 | 9.52E-01 | 1.03E+00 | 5.77E-01 | 2.50E-02 | 6 |
| rs9557207 | G | A | 6.90E-03 | 3.70E-03 | 1.00E+00 | 9.90E-01 | 1.02E+00 | 5.93E-01 | 2.09E-01 | 13 |
| rs559928 | C | T | 7.30E-03 | 3.80E-03 | 1.00E+00 | 9.90E-01 | 1.02E+00 | 5.98E-01 | 8.09E-01 | 11 |
| rs62037363 | C | T | 6.20E-03 | -3.20E-03 | 9.97E-01 | 9.85E-01 | 1.01E+00 | 6.03E-01 | 3.91E-01 | 16 |
| rs7773324 | A | G | 6.50E-03 | -3.30E-03 | 9.97E-01 | 9.84E-01 | 1.01E+00 | 6.09E-01 | 5.64E-01 | 6 |
| rs6584281 | G | A | 5.70E-03 | -2.90E-03 | 9.97E-01 | 9.86E-01 | 1.01E+00 | 6.11E-01 | 5.23E-01 | 10 |
| rs13204742 | T | G | 8.60E-03 | 4.40E-03 | 1.00E+00 | 9.88E-01 | 1.02E+00 | 6.11E-01 | 1.21E-01 | 6 |
| rs9889296 | A | G | 6.30E-03 | 3.10E-03 | 1.00E+00 | 9.91E-01 | 1.02E+00 | 6.23E-01 | 3.04E-01 | 17 |
| rs6740462 | A | C | 6.50E-03 | 3.10E-03 | 1.00E+00 | 9.90E-01 | 1.02E+00 | 6.32E-01 | 7.50E-01 | 2 |
| rs9457247 | T | C | 5.70E-03 | -2.60E-03 | 9.97E-01 | 9.86E-01 | 1.01E+00 | 6.45E-01 | 5.06E-01 | 6 |
| rs55808324 | A | G | 9.10E-03 | 4.10E-03 | 1.00E+00 | 9.86E-01 | 1.02E+00 | 6.56E-01 | 1.26E-01 | 14 |
| rs11793497 | G | A | 5.80E-03 | -2.50E-03 | 9.98E-01 | 9.86E-01 | 1.01E+00 | 6.70E-01 | 4.13E-01 | 9 |
| rs7523442 | T | C | 5.70E-03 | 2.40E-03 | 1.00E+00 | 9.91E-01 | 1.01E+00 | 6.74E-01 | 5.40E-01 | 1 |
| rs11758694 | A | T | 9.90E-03 | -4.00E-03 | 9.96E-01 | 9.77E-01 | 1.02E+00 | 6.88E-01 | 1.02E-01 | 6 |
| rs10956252 | G | C | 5.80E-03 | -2.10E-03 | 9.98E-01 | 9.87E-01 | 1.01E+00 | 7.23E-01 | 5.90E-01 | 8 |
| rs2328546 | C | T | 7.00E-03 | 2.40E-03 | 1.00E+00 | 9.89E-01 | 1.02E+00 | 7.30E-01 | 8.00E-01 | 6 |
| rs13407913 | G | A | 5.70E-03 | 2.00E-03 | 1.00E+00 | 9.91E-01 | 1.01E+00 | 7.34E-01 | 4.50E-01 | 2 |
| rs2153283 | A | C | 6.80E-03 | -2.20E-03 | 9.98E-01 | 9.85E-01 | 1.01E+00 | 7.42E-01 | 2.46E-01 | 10 |
| rs10761659 | G | A | 5.80E-03 | -1.90E-03 | 9.98E-01 | 9.87E-01 | 1.01E+00 | 7.47E-01 | 5.24E-01 | 10 |
| rs1517352 | C | A | 5.80E-03 | -1.70E-03 | 9.98E-01 | 9.87E-01 | 1.01E+00 | 7.74E-01 | 5.80E-01 | 2 |
| rs6588248 | G | T | 5.70E-03 | -1.60E-03 | 9.98E-01 | 9.87E-01 | 1.01E+00 | 7.76E-01 | 5.20E-01 | 1 |
| rs11713774 | C | T | 8.10E-03 | 1.70E-03 | 1.00E+00 | 9.86E-01 | 1.02E+00 | 8.31E-01 | 1.39E-01 | 3 |
| rs9264942 | C | T | 7.60E-03 | -1.50E-03 | 9.99E-01 | 9.84E-01 | 1.01E+00 | 8.40E-01 | 3.55E-01 | 6 |
| rs17780256 | C | A | 7.40E-03 | 1.50E-03 | 1.00E+00 | 9.87E-01 | 1.02E+00 | 8.42E-01 | 1.84E-01 | 17 |
| rs7547569 | C | T | 1.17E-02 | 2.00E-03 | 1.00E+00 | 9.79E-01 | 1.03E+00 | 8.61E-01 | 6.28E-02 | 1 |
| rs2024092 | A | G | 6.90E-03 | -1.20E-03 | 9.99E-01 | 9.85E-01 | 1.01E+00 | 8.66E-01 | 2.19E-01 | 19 |
| rs1847472 | A | C | 6.00E-03 | 1.00E-03 | 1.00E+00 | 9.89E-01 | 1.01E+00 | 8.73E-01 | 3.32E-01 | 6 |
| rs941823 | C | T | 6.60E-03 | -1.00E-03 | 9.99E-01 | 9.86E-01 | 1.01E+00 | 8.76E-01 | 7.59E-01 | 13 |
| rs2688608 | T | G | 5.70E-03 | 8.00E-04 | 1.00E+00 | 9.90E-01 | 1.01E+00 | 8.94E-01 | 5.22E-01 | 10 |
| rs11230563 | T | C | 6.00E-03 | 8.00E-04 | 1.00E+00 | 9.89E-01 | 1.01E+00 | 9.00E-01 | 3.52E-01 | 11 |
| rs2274351 | T | C | 5.70E-03 | 6.00E-04 | 1.00E+00 | 9.89E-01 | 1.01E+00 | 9.17E-01 | 5.13E-01 | 10 |
| rs1420098 | C | T | 5.90E-03 | 5.00E-04 | 1.00E+00 | 9.89E-01 | 1.01E+00 | 9.32E-01 | 3.87E-01 | 2 |
| rs1297258 | T | C | 5.70E-03 | 5.00E-04 | 1.00E+00 | 9.89E-01 | 1.01E+00 | 9.34E-01 | 4.26E-01 | 21 |
| rs34856868 | A | G | 2.14E-02 | 1.60E-03 | 1.00E+00 | 9.60E-01 | 1.04E+00 | 9.39E-01 | 2.75E-02 | 1 |
| rs6500315 | G | A | 6.90E-03 | 4.00E-04 | 1.00E+00 | 9.87E-01 | 1.01E+00 | 9.49E-01 | 7.81E-01 | 16 |
| rs1292053 | G | A | 5.70E-03 | -4.00E-04 | 1.00E+00 | 9.88E-01 | 1.01E+00 | 9.50E-01 | 4.51E-01 | 17 |
| rs11185982 | C | T | 8.00E-03 | 5.00E-04 | 1.00E+00 | 9.85E-01 | 1.02E+00 | 9.50E-01 | 1.44E-01 | 10 |
| rs79980175 | C | A | 8.30E-03 | -5.00E-04 | 1.00E+00 | 9.83E-01 | 1.02E+00 | 9.56E-01 | 1.36E-01 | 5 |
| rs3853824 | C | T | 5.90E-03 | 3.00E-04 | 1.00E+00 | 9.89E-01 | 1.01E+00 | 9.57E-01 | 6.49E-01 | 17 |
| rs10878302 | A | T | 1.05E-02 | 5.00E-04 | 1.00E+00 | 9.80E-01 | 1.02E+00 | 9.63E-01 | 9.14E-01 | 12 |
| rs4795397 | G | A | 5.80E-03 | -2.00E-04 | 1.00E+00 | 9.88E-01 | 1.01E+00 | 9.69E-01 | 4.54E-01 | 17 |
| rs11677953 | A | G | 5.80E-03 | -2.00E-04 | 1.00E+00 | 9.88E-01 | 1.01E+00 | 9.70E-01 | 3.96E-01 | 2 |
| rs11691685 | G | A | 1.06E-02 | -4.00E-04 | 1.00E+00 | 9.79E-01 | 1.02E+00 | 9.70E-01 | 7.54E-02 | 2 |
| rs974801 | G | A | 5.80E-03 | -2.00E-04 | 1.00E+00 | 9.88E-01 | 1.01E+00 | 9.71E-01 | 3.74E-01 | 4 |
| rs1363907 | A | G | 5.80E-03 | -2.00E-04 | 1.00E+00 | 9.88E-01 | 1.01E+00 | 9.75E-01 | 4.13E-01 | 5 |
| rs1250566 | A | G | 6.30E-03 | 2.00E-04 | 1.00E+00 | 9.88E-01 | 1.01E+00 | 9.79E-01 | 3.00E-01 | 10 |
| rs10800309 | G | A | 6.00E-03 | -1.00E-04 | 1.00E+00 | 9.88E-01 | 1.01E+00 | 9.80E-01 | 6.65E-01 | 1 |
| rs13107612 | T | C | 6.20E-03 | -1.00E-04 | 1.00E+00 | 9.88E-01 | 1.01E+00 | 9.88E-01 | 2.98E-01 | 4 |

**Supplementary Table8.** Detailed information of SNPs acting as Instrumental variants (IVs) in MR analysis of IBD on total body BMD. This list was ordered by P value of each SNP associated with total body BMD. OR: odds ratio; lo_CI: lower bound 95% confidence interval of odds ratio; up_CI: upper bound 95% confidence interval of odds ratio; chr: chromosome; se: standard error; eaf: effect allele frequency.

| **SNP** | **effect allele** | **other allele** | **se** | **beta** | **OR** | **lo_CI** | **up_CI** | **pval** | **eaf** | **chr** |
| --- | --- | --- | --- | --- | --- | --- | --- | --- | --- | --- |
| rs34779708 | G | T | 5.39E-02 | -1.74E-01 | 8.40E-01 | 7.56E-01 | 9.34E-01 | 1.24E-03 | 3.73E-01 | 10 |
| rs4703855 | T | C | 5.40E-02 | -1.49E-01 | 8.61E-01 | 7.75E-01 | 9.58E-01 | 5.70E-03 | 3.67E-01 | 5 |
| rs56167332 | A | C | 5.64E-02 | 1.30E-01 | 1.14E+00 | 1.02E+00 | 1.27E+00 | 2.17E-02 | 3.06E-01 | 5 |
| rs913678 | C | T | 5.50E-02 | 1.21E-01 | 1.13E+00 | 1.01E+00 | 1.26E+00 | 2.78E-02 | 3.50E-01 | 20 |
| rs6933404 | C | T | 6.64E-02 | 1.36E-01 | 1.15E+00 | 1.01E+00 | 1.30E+00 | 4.08E-02 | 1.88E-01 | 6 |
| rs1517352 | C | A | 5.40E-02 | -1.05E-01 | 9.00E-01 | 8.10E-01 | 1.00E+00 | 5.15E-02 | 6.25E-01 | 2 |
| rs6708373 | G | A | 5.27E-02 | 9.98E-02 | 1.10E+00 | 9.97E-01 | 1.23E+00 | 5.82E-02 | 4.40E-01 | 2 |
| rs6584281 | G | A | 5.21E-02 | -9.57E-02 | 9.09E-01 | 8.21E-01 | 1.01E+00 | 6.63E-02 | 5.30E-01 | 10 |
| rs6651252 | C | T | 8.19E-02 | -1.45E-01 | 8.65E-01 | 7.37E-01 | 1.02E+00 | 7.60E-02 | 1.16E-01 | 8 |
| rs34804116 | A | C | 5.38E-02 | -9.39E-02 | 9.10E-01 | 8.19E-01 | 1.01E+00 | 8.08E-02 | 3.67E-01 | 5 |
| rs11677953 | A | G | 5.65E-02 | -9.58E-02 | 9.09E-01 | 8.13E-01 | 1.02E+00 | 9.01E-02 | 3.06E-01 | 2 |
| rs1363907 | A | G | 5.36E-02 | -9.00E-02 | 9.14E-01 | 8.23E-01 | 1.02E+00 | 9.28E-02 | 3.79E-01 | 5 |
| rs17293632 | T | C | 5.95E-02 | 9.76E-02 | 1.10E+00 | 9.81E-01 | 1.24E+00 | 1.01E-01 | 2.62E-01 | 15 |
| rs2328546 | C | T | 6.03E-02 | -9.80E-02 | 9.07E-01 | 8.06E-01 | 1.02E+00 | 1.04E-01 | 7.54E-01 | 6 |
| rs1182188 | C | T | 5.46E-02 | -8.72E-02 | 9.16E-01 | 8.23E-01 | 1.02E+00 | 1.10E-01 | 3.61E-01 | 7 |
| rs140143 | T | G | 5.45E-02 | 8.68E-02 | 1.09E+00 | 9.80E-01 | 1.21E+00 | 1.12E-01 | 3.48E-01 | 22 |
| rs11793497 | G | A | 5.29E-02 | -8.09E-02 | 9.22E-01 | 8.31E-01 | 1.02E+00 | 1.26E-01 | 4.17E-01 | 9 |
| rs11185982 | C | T | 7.96E-02 | 1.12E-01 | 1.12E+00 | 9.57E-01 | 1.31E+00 | 1.59E-01 | 1.23E-01 | 10 |
| rs10758669 | A | C | 5.43E-02 | -7.57E-02 | 9.27E-01 | 8.33E-01 | 1.03E+00 | 1.63E-01 | 6.42E-01 | 9 |
| rs2538470 | G | A | 5.37E-02 | -7.44E-02 | 9.28E-01 | 8.36E-01 | 1.03E+00 | 1.66E-01 | 6.18E-01 | 7 |
| rs10142466 | G | A | 5.21E-02 | -7.08E-02 | 9.32E-01 | 8.41E-01 | 1.03E+00 | 1.74E-01 | 4.88E-01 | 14 |
| rs6500315 | G | A | 7.31E-02 | 9.82E-02 | 1.10E+00 | 9.56E-01 | 1.27E+00 | 1.79E-01 | 8.48E-01 | 16 |
| rs17651741 | A | G | 6.58E-02 | -8.62E-02 | 9.17E-01 | 8.06E-01 | 1.04E+00 | 1.91E-01 | 1.97E-01 | 15 |
| rs2050392 | A | G | 5.30E-02 | 6.79E-02 | 1.07E+00 | 9.65E-01 | 1.19E+00 | 2.00E-01 | 5.88E-01 | 10 |
| rs2836883 | A | G | 6.07E-02 | -7.34E-02 | 9.29E-01 | 8.25E-01 | 1.05E+00 | 2.26E-01 | 2.48E-01 | 21 |
| rs3776414 | G | T | 5.71E-02 | -6.56E-02 | 9.37E-01 | 8.37E-01 | 1.05E+00 | 2.51E-01 | 2.98E-01 | 5 |
| rs11236797 | A | C | 5.28E-02 | 5.95E-02 | 1.06E+00 | 9.57E-01 | 1.18E+00 | 2.60E-01 | 4.07E-01 | 11 |
| rs974801 | G | A | 5.35E-02 | 5.98E-02 | 1.06E+00 | 9.56E-01 | 1.18E+00 | 2.64E-01 | 3.84E-01 | 4 |
| rs7547569 | C | T | 1.24E-01 | -1.37E-01 | 8.72E-01 | 6.84E-01 | 1.11E+00 | 2.70E-01 | 4.79E-02 | 1 |
| rs259964 | G | A | 5.29E-02 | 5.80E-02 | 1.06E+00 | 9.55E-01 | 1.18E+00 | 2.73E-01 | 5.77E-01 | 20 |
| rs516246 | T | C | 5.37E-02 | 5.87E-02 | 1.06E+00 | 9.55E-01 | 1.18E+00 | 2.74E-01 | 3.75E-01 | 19 |
| rs6456426 | A | C | 5.21E-02 | -5.67E-02 | 9.45E-01 | 8.53E-01 | 1.05E+00 | 2.76E-01 | 4.86E-01 | 6 |
| rs6561151 | A | G | 6.46E-02 | 6.96E-02 | 1.07E+00 | 9.45E-01 | 1.22E+00 | 2.82E-01 | 2.00E-01 | 13 |
| rs71593329 | G | T | 7.35E-02 | 7.85E-02 | 1.08E+00 | 9.37E-01 | 1.25E+00 | 2.86E-01 | 1.52E-01 | 5 |
| rs10878302 | A | T | 1.09E-01 | -1.16E-01 | 8.91E-01 | 7.20E-01 | 1.10E+00 | 2.89E-01 | 9.40E-01 | 12 |
| rs13407913 | G | A | 5.30E-02 | 5.55E-02 | 1.06E+00 | 9.53E-01 | 1.17E+00 | 2.96E-01 | 4.08E-01 | 2 |
| rs2688608 | T | G | 5.22E-02 | -5.33E-02 | 9.48E-01 | 8.56E-01 | 1.05E+00 | 3.07E-01 | 4.69E-01 | 10 |
| rs6111031 | T | C | 7.82E-02 | -7.72E-02 | 9.26E-01 | 7.94E-01 | 1.08E+00 | 3.23E-01 | 1.26E-01 | 20 |
| rs2974935 | T | G | 5.23E-02 | 5.06E-02 | 1.05E+00 | 9.49E-01 | 1.17E+00 | 3.33E-01 | 5.43E-01 | 1 |
| rs62434177 | A | G | 1.13E-01 | 1.09E-01 | 1.12E+00 | 8.94E-01 | 1.39E+00 | 3.35E-01 | 5.89E-02 | 6 |
| rs2395022 | C | A | 1.27E-01 | -1.21E-01 | 8.86E-01 | 6.91E-01 | 1.13E+00 | 3.37E-01 | 9.56E-01 | 7 |
| rs4692386 | C | T | 5.40E-02 | 5.16E-02 | 1.05E+00 | 9.47E-01 | 1.17E+00 | 3.39E-01 | 6.22E-01 | 4 |
| rs181826 | A | C | 5.50E-02 | 5.20E-02 | 1.05E+00 | 9.46E-01 | 1.17E+00 | 3.44E-01 | 6.56E-01 | 5 |
| rs6058869 | T | C | 5.31E-02 | 4.92E-02 | 1.05E+00 | 9.47E-01 | 1.17E+00 | 3.54E-01 | 4.02E-01 | 20 |
| rs1388585 | A | G | 1.63E-01 | 1.51E-01 | 1.16E+00 | 8.45E-01 | 1.60E+00 | 3.55E-01 | 9.73E-01 | 12 |
| rs3024493 | A | C | 7.16E-02 | 6.57E-02 | 1.07E+00 | 9.28E-01 | 1.23E+00 | 3.59E-01 | 1.56E-01 | 1 |
| rs35164067 | A | G | 6.84E-02 | -6.17E-02 | 9.40E-01 | 8.22E-01 | 1.08E+00 | 3.68E-01 | 1.76E-01 | 19 |
| rs3184504 | C | T | 5.28E-02 | -4.72E-02 | 9.54E-01 | 8.60E-01 | 1.06E+00 | 3.71E-01 | 5.92E-01 | 12 |
| rs55808324 | A | G | 9.57E-02 | 8.49E-02 | 1.09E+00 | 9.02E-01 | 1.31E+00 | 3.75E-01 | 8.22E-02 | 14 |
| rs4795397 | G | A | 5.23E-02 | 4.35E-02 | 1.04E+00 | 9.43E-01 | 1.16E+00 | 4.05E-01 | 5.18E-01 | 17 |
| rs6588248 | G | T | 5.19E-02 | -4.27E-02 | 9.58E-01 | 8.66E-01 | 1.06E+00 | 4.11E-01 | 4.99E-01 | 1 |
| rs6745185 | G | T | 6.97E-02 | -5.65E-02 | 9.45E-01 | 8.24E-01 | 1.08E+00 | 4.17E-01 | 8.34E-01 | 2 |
| rs7848647 | C | T | 5.61E-02 | 4.50E-02 | 1.05E+00 | 9.37E-01 | 1.17E+00 | 4.23E-01 | 6.94E-01 | 9 |
| rs13204742 | T | G | 7.71E-02 | -6.08E-02 | 9.41E-01 | 8.09E-01 | 1.09E+00 | 4.30E-01 | 1.30E-01 | 6 |
| rs559928 | C | T | 6.50E-02 | 5.05E-02 | 1.05E+00 | 9.26E-01 | 1.19E+00 | 4.37E-01 | 8.01E-01 | 11 |
| rs11691685 | G | A | 9.69E-02 | 7.51E-02 | 1.08E+00 | 8.92E-01 | 1.30E+00 | 4.38E-01 | 7.82E-02 | 2 |
| rs6466198 | T | A | 5.38E-02 | 4.12E-02 | 1.04E+00 | 9.38E-01 | 1.16E+00 | 4.44E-01 | 3.70E-01 | 7 |
| rs1267499 | C | T | 8.35E-02 | -6.36E-02 | 9.38E-01 | 7.97E-01 | 1.11E+00 | 4.46E-01 | 8.88E-01 | 6 |
| rs12796489 | A | C | 3.97E-01 | -3.03E-01 | 7.39E-01 | 3.39E-01 | 1.61E+00 | 4.46E-01 | 4.28E-03 | 11 |
| rs2488397 | C | G | 7.42E-02 | -5.60E-02 | 9.46E-01 | 8.18E-01 | 1.09E+00 | 4.50E-01 | 1.46E-01 | 1 |
| rs35730213 | C | G | 6.36E-02 | 4.79E-02 | 1.05E+00 | 9.26E-01 | 1.19E+00 | 4.51E-01 | 2.13E-01 | 1 |
| rs1250566 | A | G | 5.32E-02 | -3.97E-02 | 9.61E-01 | 8.66E-01 | 1.07E+00 | 4.56E-01 | 4.10E-01 | 10 |
| rs6062496 | A | G | 5.42E-02 | 3.99E-02 | 1.04E+00 | 9.36E-01 | 1.16E+00 | 4.62E-01 | 6.44E-01 | 20 |
| rs7194886 | T | C | 5.21E-02 | -3.82E-02 | 9.63E-01 | 8.69E-01 | 1.07E+00 | 4.63E-01 | 4.89E-01 | 16 |
| rs13107612 | T | C | 5.64E-02 | 4.09E-02 | 1.04E+00 | 9.33E-01 | 1.16E+00 | 4.69E-01 | 3.10E-01 | 4 |
| rs1420098 | C | T | 5.34E-02 | -3.78E-02 | 9.63E-01 | 8.67E-01 | 1.07E+00 | 4.80E-01 | 4.01E-01 | 2 |
| rs9264942 | C | T | 5.87E-02 | -4.10E-02 | 9.60E-01 | 8.56E-01 | 1.08E+00 | 4.85E-01 | 2.71E-01 | 6 |
| rs10800309 | G | A | 5.37E-02 | -3.76E-02 | 9.63E-01 | 8.67E-01 | 1.07E+00 | 4.85E-01 | 6.29E-01 | 1 |
| rs9557207 | G | A | 7.68E-02 | -5.36E-02 | 9.48E-01 | 8.15E-01 | 1.10E+00 | 4.85E-01 | 1.32E-01 | 13 |
| rs36048684 | A | T | 1.03E-01 | 7.06E-02 | 1.07E+00 | 8.77E-01 | 1.31E+00 | 4.92E-01 | 7.06E-02 | 5 |
| rs3853824 | C | T | 5.28E-02 | -3.60E-02 | 9.65E-01 | 8.70E-01 | 1.07E+00 | 4.95E-01 | 5.60E-01 | 17 |
| rs9457247 | T | C | 5.21E-02 | 3.51E-02 | 1.04E+00 | 9.35E-01 | 1.15E+00 | 5.00E-01 | 4.88E-01 | 6 |
| rs780094 | C | T | 5.47E-02 | 3.61E-02 | 1.04E+00 | 9.31E-01 | 1.15E+00 | 5.09E-01 | 6.44E-01 | 2 |
| rs72924296 | G | A | 6.23E-02 | -3.97E-02 | 9.61E-01 | 8.51E-01 | 1.09E+00 | 5.24E-01 | 2.26E-01 | 2 |
| rs11152949 | G | A | 6.05E-02 | 3.83E-02 | 1.04E+00 | 9.23E-01 | 1.17E+00 | 5.27E-01 | 2.46E-01 | 6 |
| rs7608910 | G | A | 5.38E-02 | -3.09E-02 | 9.70E-01 | 8.73E-01 | 1.08E+00 | 5.65E-01 | 3.69E-01 | 2 |
| rs2143178 | C | T | 8.04E-02 | 4.56E-02 | 1.05E+00 | 8.94E-01 | 1.23E+00 | 5.70E-01 | 1.17E-01 | 22 |
| rs62037363 | C | T | 5.26E-02 | 2.96E-02 | 1.03E+00 | 9.29E-01 | 1.14E+00 | 5.74E-01 | 4.12E-01 | 16 |
| rs744166 | G | A | 5.28E-02 | -2.96E-02 | 9.71E-01 | 8.75E-01 | 1.08E+00 | 5.75E-01 | 4.21E-01 | 17 |
| rs9836291 | A | G | 5.31E-02 | 2.94E-02 | 1.03E+00 | 9.28E-01 | 1.14E+00 | 5.79E-01 | 4.13E-01 | 3 |
| rs12585310 | A | G | 5.60E-02 | 3.06E-02 | 1.03E+00 | 9.24E-01 | 1.15E+00 | 5.85E-01 | 3.21E-01 | 13 |
| rs1297258 | T | C | 5.28E-02 | 2.82E-02 | 1.03E+00 | 9.27E-01 | 1.14E+00 | 5.94E-01 | 4.05E-01 | 21 |
| rs1847472 | A | C | 6.01E-02 | -3.05E-02 | 9.70E-01 | 8.62E-01 | 1.09E+00 | 6.12E-01 | 2.51E-01 | 6 |
| rs11768997 | T | G | 1.22E-01 | 5.86E-02 | 1.06E+00 | 8.35E-01 | 1.35E+00 | 6.31E-01 | 5.07E-02 | 7 |
| rs367569 | T | C | 5.83E-02 | -2.78E-02 | 9.73E-01 | 8.68E-01 | 1.09E+00 | 6.34E-01 | 2.76E-01 | 16 |
| rs12411259 | A | G | 5.80E-02 | -2.67E-02 | 9.74E-01 | 8.69E-01 | 1.09E+00 | 6.46E-01 | 2.83E-01 | 1 |
| rs9889296 | A | G | 5.51E-02 | -2.45E-02 | 9.76E-01 | 8.76E-01 | 1.09E+00 | 6.56E-01 | 3.36E-01 | 17 |
| rs2153283 | A | C | 6.13E-02 | -2.55E-02 | 9.75E-01 | 8.64E-01 | 1.10E+00 | 6.77E-01 | 2.36E-01 | 10 |
| rs67643815 | T | G | 5.26E-02 | -2.14E-02 | 9.79E-01 | 8.83E-01 | 1.09E+00 | 6.84E-01 | 5.86E-01 | 18 |
| rs12722515 | A | C | 7.48E-02 | 2.96E-02 | 1.03E+00 | 8.90E-01 | 1.19E+00 | 6.93E-01 | 1.44E-01 | 10 |
| rs10761659 | G | A | 5.20E-02 | 2.05E-02 | 1.02E+00 | 9.22E-01 | 1.13E+00 | 6.94E-01 | 5.29E-01 | 10 |
| rs7015630 | C | T | 5.87E-02 | 2.05E-02 | 1.02E+00 | 9.10E-01 | 1.15E+00 | 7.26E-01 | 2.71E-01 | 8 |
| rs2274351 | T | C | 5.21E-02 | -1.81E-02 | 9.82E-01 | 8.87E-01 | 1.09E+00 | 7.28E-01 | 5.22E-01 | 10 |
| rs34856868 | A | G | 1.44E-01 | -4.99E-02 | 9.51E-01 | 7.18E-01 | 1.26E+00 | 7.29E-01 | 3.36E-02 | 1 |
| rs9273363 | A | C | 6.20E-02 | 2.10E-02 | 1.02E+00 | 9.04E-01 | 1.15E+00 | 7.35E-01 | 2.79E-01 | 6 |
| rs1569328 | T | C | 6.77E-02 | -2.16E-02 | 9.79E-01 | 8.57E-01 | 1.12E+00 | 7.49E-01 | 1.79E-01 | 14 |
| rs72634258 | C | T | 6.00E-02 | -1.89E-02 | 9.81E-01 | 8.72E-01 | 1.10E+00 | 7.53E-01 | 2.54E-01 | 1 |
| rs272882 | T | G | 5.30E-02 | 1.63E-02 | 1.02E+00 | 9.16E-01 | 1.13E+00 | 7.59E-01 | 5.86E-01 | 5 |
| rs1292053 | G | A | 5.27E-02 | 1.60E-02 | 1.02E+00 | 9.16E-01 | 1.13E+00 | 7.61E-01 | 4.34E-01 | 17 |
| rs12718244 | A | G | 5.73E-02 | -1.70E-02 | 9.83E-01 | 8.79E-01 | 1.10E+00 | 7.67E-01 | 2.93E-01 | 7 |
| rs1990760 | T | C | 5.27E-02 | -1.53E-02 | 9.85E-01 | 8.88E-01 | 1.09E+00 | 7.71E-01 | 5.85E-01 | 2 |
| rs11713774 | C | T | 8.10E-02 | -2.33E-02 | 9.77E-01 | 8.34E-01 | 1.15E+00 | 7.73E-01 | 1.18E-01 | 3 |
| rs2297559 | A | G | 5.56E-02 | 1.58E-02 | 1.02E+00 | 9.11E-01 | 1.13E+00 | 7.76E-01 | 6.79E-01 | 1 |
| rs769177 | T | C | 1.13E-01 | 3.21E-02 | 1.03E+00 | 8.27E-01 | 1.29E+00 | 7.76E-01 | 5.70E-02 | 6 |
| rs2270395 | T | C | 5.65E-02 | 1.57E-02 | 1.02E+00 | 9.09E-01 | 1.13E+00 | 7.82E-01 | 6.95E-01 | 16 |
| rs35256947 | C | T | 6.30E-02 | -1.72E-02 | 9.83E-01 | 8.69E-01 | 1.11E+00 | 7.85E-01 | 2.17E-01 | 2 |
| rs3801835 | T | C | 5.82E-02 | -1.56E-02 | 9.85E-01 | 8.78E-01 | 1.10E+00 | 7.89E-01 | 2.77E-01 | 7 |
| rs10956252 | G | C | 5.81E-02 | 1.52E-02 | 1.02E+00 | 9.06E-01 | 1.14E+00 | 7.94E-01 | 7.23E-01 | 8 |
| rs11641016 | G | C | 5.94E-02 | -1.50E-02 | 9.85E-01 | 8.77E-01 | 1.11E+00 | 8.01E-01 | 2.66E-01 | 16 |
| rs2847278 | T | C | 7.43E-02 | 1.71E-02 | 1.02E+00 | 8.79E-01 | 1.18E+00 | 8.18E-01 | 8.53E-01 | 18 |
| rs17694108 | A | G | 5.49E-02 | -1.24E-02 | 9.88E-01 | 8.87E-01 | 1.10E+00 | 8.21E-01 | 3.48E-01 | 19 |
| rs7011507 | A | G | 9.50E-02 | 2.14E-02 | 1.02E+00 | 8.48E-01 | 1.23E+00 | 8.22E-01 | 8.38E-02 | 8 |
| rs648541 | G | A | 5.32E-02 | -1.14E-02 | 9.89E-01 | 8.91E-01 | 1.10E+00 | 8.31E-01 | 3.92E-01 | 11 |
| rs7657746 | G | A | 7.50E-02 | 1.41E-02 | 1.01E+00 | 8.76E-01 | 1.17E+00 | 8.51E-01 | 1.40E-01 | 4 |
| rs6074022 | T | C | 5.82E-02 | -1.08E-02 | 9.89E-01 | 8.83E-01 | 1.11E+00 | 8.53E-01 | 7.27E-01 | 20 |
| rs78487399 | C | G | 1.13E-01 | -1.96E-02 | 9.81E-01 | 7.86E-01 | 1.22E+00 | 8.62E-01 | 9.45E-01 | 2 |
| rs2024092 | A | G | 6.18E-02 | -1.05E-02 | 9.90E-01 | 8.77E-01 | 1.12E+00 | 8.65E-01 | 2.32E-01 | 19 |
| rs941823 | C | T | 5.89E-02 | 1.00E-02 | 1.01E+00 | 9.00E-01 | 1.13E+00 | 8.65E-01 | 7.29E-01 | 13 |
| rs7523442 | T | C | 5.23E-02 | 8.80E-03 | 1.01E+00 | 9.11E-01 | 1.12E+00 | 8.66E-01 | 4.92E-01 | 1 |
| rs4976646 | C | T | 5.44E-02 | 8.90E-03 | 1.01E+00 | 9.07E-01 | 1.12E+00 | 8.69E-01 | 3.57E-01 | 5 |
| rs12318183 | A | C | 5.57E-02 | -9.10E-03 | 9.91E-01 | 8.88E-01 | 1.11E+00 | 8.70E-01 | 3.23E-01 | 12 |
| rs7253253 | T | G | 1.11E-01 | -1.69E-02 | 9.83E-01 | 7.91E-01 | 1.22E+00 | 8.79E-01 | 9.41E-01 | 19 |
| rs4743820 | T | C | 5.51E-02 | 8.10E-03 | 1.01E+00 | 9.05E-01 | 1.12E+00 | 8.83E-01 | 6.66E-01 | 9 |
| rs7240004 | G | A | 5.41E-02 | -6.80E-03 | 9.93E-01 | 8.93E-01 | 1.10E+00 | 9.00E-01 | 3.78E-01 | 18 |
| rs17780256 | C | A | 6.93E-02 | 8.40E-03 | 1.01E+00 | 8.80E-01 | 1.16E+00 | 9.04E-01 | 1.70E-01 | 17 |
| rs11230563 | T | C | 6.04E-02 | -5.40E-03 | 9.95E-01 | 8.84E-01 | 1.12E+00 | 9.28E-01 | 2.49E-01 | 11 |
| rs2497318 | T | C | 5.24E-02 | -4.00E-03 | 9.96E-01 | 8.99E-01 | 1.10E+00 | 9.39E-01 | 4.49E-01 | 10 |
| rs7773324 | A | G | 5.26E-02 | 3.70E-03 | 1.00E+00 | 9.05E-01 | 1.11E+00 | 9.44E-01 | 5.52E-01 | 6 |
| rs6740462 | A | C | 5.41E-02 | 2.50E-03 | 1.00E+00 | 9.02E-01 | 1.11E+00 | 9.62E-01 | 6.26E-01 | 2 |

**Supplementary Table9.** Detailed information of SNPs acting as Instrumental variants (IVs) in MR analysis of IBD on OS with FG. This list was ordered by P value of each SNP associated with OS with FG. OR: odds ratio; lo_CI: lower bound 95% confidence interval of odds ratio; up_CI: upper bound 95% confidence interval of odds ratio; chr: chromosome; se: standard error; eaf: effect allele frequency.
